# Comprehensive genome-wide identification and transferability of chromosome-specific highly variable microsatellite markers from citrus species

**DOI:** 10.1038/s41598-023-37024-0

**Published:** 2023-07-05

**Authors:** Jagveer Singh, Ankush Sharma, Vishal Sharma, Popat Nanaso Gaikwad, Gurupkar Singh Sidhu, Gurwinder Kaur, Nimarpreet Kaur, Taveena Jindal, Parveen Chhuneja, H. S. Rattanpal

**Affiliations:** 1 School of Agricultural Biotechnology, Punjab Agricultural University, Ludhiana, 141004 India; 2 Department of Fruit Science, College of Horticulture & Forestry, Acharya Narendra Deva University of Agricultural & Technology, Kumarganj, 224229 India; 3 Plant Genome Mapping Laboratory, University of Georgia, Athens, GA 30602 USA; 4 National Agri-Food Biotechnology Institute, Sector-81, SAS Nagar, Mohali, Punjab 140308 India; 5 Faculty of Applied Sciences and Biotechnology, Shoolini University, Solan, 173229 India; 6 Department of Fruit Science, Punjab Agricultural University, Ludhiana, 141004 India

**Keywords:** Biotechnology, Computational biology and bioinformatics, Genetics, Molecular biology, Plant sciences

## Abstract

Citrus species among the most important and widely consumed fruit in the world due to Vitamin C, essential oil glands, and flavonoids. Highly variable simple sequence repeats (SSR) markers are one of the most informative and versatile molecular markers used in perennial tree genetic research. SSR survey of *Citrus sinensis* and *Citrus maxima* were identified perfect SSRs spanning nine chromosomes. Furthermore, we categorized all SSR motifs into three major classes based on their tract lengths. We designed and validated a class I SSRs in the *C. sinensis* and *C. maxima* genome through electronic polymerase chain reaction (ePCR) and found 83.89% in *C. sinensis* and 78.52% in *C. maxima* SSRs producing a single amplicon. Then, we selected **extremely variable** SSRs (> 40 nt) from the ePCR-verified class I SSRs and in silico validated across seven draft genomes of citrus, which provided us a subset of 84.74% in *C. sinensis* and 77.53% in *C. maxima* highly polymorphic SSRs. Out of these, 129 primers were validated on 24 citrus genotypes through wet-lab experiment. We found 127 (98.45%) polymorphic HvSSRs on 24 genotypes. The utility of the developed HvSSRs was demonstrated by analysing genetic diversity of 181 citrus genotypes using 17 HvSSRs spanning nine citrus chromosomes and were divided into 11 main groups through 17 HvSSRs. These chromosome-specific SSRs will serve as a powerful genomic tool used for future QTL mapping, molecular breeding, investigation of population genetic diversity, comparative mapping, and evolutionary studies among citrus and other relative genera/species.

## Introduction

Citrus and allied genera with essential oil glands (*Eremocitrus*, *Fortunella*, *Microcitrus*, *Clymenia*, and *Poncirus*) are members of the Rutaceae family. Citrus was classified by Tanaka^1^, Swingle and Reece^2^, and Mabberley^3^ revised by Zhang and Mabberley^4^. Citrus is produced throughout the monsoon region from more than 145 countries in the world. In 2020, the production of citrus fruit reached 158 million tons worldwide^5^. It is a popular fruit crop that is recognised for its energising scent, high vitamin C content, and health-promoting properties^6^. Therefore, citrus fruits are in high demand all around the world^7^. Citrus taxonomy is complicated by the peculiar manner of reproduction, a high frequency of bud mutations, apomictic, and the species extensive cross-compatibility^7–10^. However, on the other hand conventional citrus breeding takes at least 3–8 years to complete due to the lengthy juvenile stage^2^.

Using molecular breeding, pre-selection from a large number of different individuals to discover and introduce precise genetic sequences that can impart desired attributes (precocious bearing, nutritional quality improvement, resistance to biotic and abiotic stresses) is an effective strategy for improving scion and rootstock^11,12^. Microsatellites are widely used, prolific, and convenient for genomic/genetic and molecular breeding studies^13–17^.

SSRs are tandem repetitions of 1–6 nucleotides of DNA flanked by unique sequences found mostly in the intronic region of the genome, showing multi-allelic variation, excellent repeatability, co-dominant inheritance^18,19^, highly versatile, low-cost, highly informative PCR-based marker associated with a high frequency polymorphism^20^. SSR mutation rates range from 10^−3^ to 10^−6^ per cell generation, and rise as the repeat unit length increases^21,22^. These characteristics distinguish SSRs from other genetic markers such as AFLP, RAPD, RFLP, SNP, and SRAP and lay the groundwork for their use in genetic mapping, QTL identification, varietal identification, marker-assisted selection, and evolutionary research^23^. Biswas et al.^24^ were identified two classes of SSRs in the *Citrus sinensis* genome^25^: 20 bp total length in Class I and 16–19 bp total length in Class II. On the basis of length and repetitions, three primary groups of SSRs were identified in brinjal and pomegranate: class I (hypervariable: > 30 nt) and **extremely variable** SSRs (> 40 nt), class II (possibly variable: 20–30 nt), and class III (variable: 20 nt)^17,26^. In several crops, the importance of SSR marker tract lengths used for marker design and marker-assisted breeding has been proven^17,24,27–30^. Available genomic sequences which help to develop of large-scale molecular markers which spanning the whole genome like SSRs gives the information for attributes discovery and molecular breeding. Comprehensive genome-wide SSR markers were successfully implemented in many perennial plant species viz*.,* Grape^31^, Citrus^24^, Chinese jujube^32^, Prunus^33^, Olive^34^ Eggplant^26^, Banana^35^, and Pomegranate^17,36^.

SSR markers are being used widely in citrus, for assessment of genetic variability, association studies, and population structure^9,37–44^. The most of these investigations, found low level of variability. The lack of highly variable and chromosome specific DNA markers has impeded the development of highly saturated genetic linkage maps for QTL mapping and marker-assisted selection. The genome-wide characterisation and production of the first set of chromosome-specific highly polymorphic SSR markers in citrus was made possible by chromosome-level assembly of citrus species such as *Citrus sinensis* (L.) Osbeck^25^ and *Citrus maxima*^45^. *Citrus sinensis* is the most worldwide important citrus scion species. Sweet orange juice is characterized by sweet and pleasant taste, a fine aroma, ascorbic acid and hesperidin that is much appreciated by consumers. Pummelo (*Citrus maxima*) is described as the largest citrus fruit. It is often served halved and sprinkled with sugar. It is also a good source of vitamin C, iron, potassium, and calcium. It is used as germplasm to transfer genes of interest in the elite scion and rootstocks. Hence, it was hypothesised that *C. sinensis* and *C. maxima* can be utilized as model genotypes in citrus genomic research.

In view of this, the present study was aimed; (1) *In-silico* Genome wide comprehensive chromosome wise SSR development and validation of *C. sinensis* and *C. maxima*. (2) Population structure and genetic variability analysis of citrus germplasms (181) on the basis chromosome wise highly variable SSR markers. The present results could be the vast utility for the citriculture and molecular breeding viz., closely related cultivar identification, Genome-wide association studies (GWAS), Marker Assisted Selection (MAS).

## Results

### Genome-wide discovery of SSRs

Genome-wide 1,08,833 and 1,29,321 perfect SSRs were identified, compared to 494,611 and 608,896 imperfect SSRs in *C. sinensis* and *C. maxima*, respectively. The mean marker densities (SSRs/Mb) of perfect SSRs and imperfect SSRs were found to be 331.86 and 1508.21 in *C. sinensis* and 373.99 and 1760.94 in *C. maxima*, respectively (Table 1). A total of 3833 and 5042 compound SSRs were detected in *C. sinensis* and *C. maxima*, respectively. The mononucleotide repeats were most abundant than di- to hexanucleotide repeats in both citrus species. The mononucleotide and di-nucleotide repeats in *C. sinensis* were 56,355 (51.78%) and 21,436 (19.70%), respectively, while *C. maxima* genome has 71,513 (55.30%) mononucleotide repeats and 23,468 (18.15%) di-nucleotide repeats Table 2. The frequency distribution of different types of SSR motifs in the *C. sinensis* genomic sequence is depicted in Supplementary Fig. 1A,B. Among them, mono found to be highest occurrence (51.9%), thereafter di (19.6%), tri (16.7%), tetra (8.2%), penta (2.6%), and hexa (1.0%) nucleotide and the same pattern was observed in *C. maxima* (Supplementary Fig. 1C,D).

**Table 1:** Characterization of microsatellites in the *Citrus sinensis* and *Citrus maxima.*

SSR mining	*C. Sinensis*	*C. Maxima*
Examined sequences size (bp)	327,944,670	345,779,982
Total number of perfect SSR (s)	108,833	129,321
Total length of perfect SSR (bp)	2,309,993	2,307,179
Relative Abundace of SSR (loci/Mb)	361.39	374.96
Relative Density of SSR (bp/Mb)	7670.56	6689.59
Total number of compound SSR (s)	3833	5042

**Table 2:** Characterization of microsatellites in the *Citrus sinensis* and *Citrus maxima.*

Type	*C. sinensis*						*C. maxima*					
	Counts	Length (bp)	Percent (%)	Average length (bp)	Relative abundance (loci/Mb)	Relative density (bp/Mb)	Counts	Length (bp)	Percent (%)	Average length (bp)	Relative abundance (loci/Mb)	Relative density (bp/Mb)
Mono	56,355	886,852	51.78	15.74	187.13	2944.88	71,513	1,074,501	55.3	15.03	207.35	3115.48
Di	21,436	541,502	19.7	25.26	71.18	1798.11	23,468	512,592	18.15	21.84	68.04	1486.24
Tri	18,208	608,031	16.73	33.39	60.46	2019.03	20,215	440,223	15.63	21.78	58.61	1276.41
Tetra	8877	180,032	8.16	20.28	29.48	597.81	9560	174,820	7.39	18.29	27.72	506.89
Penta	2816	60,570	2.59	21.51	9.35	201.13	3119	67,045	2.41	21.5	9.04	194.39
Hexa	1141	33,006	1.05	28.93	3.79	109.6	1446	37,998	1.12	26.28	4.19	110.17

Among the mononucleotide to hexanucleotide repeats, the most motifs were found like ‘A’, ‘AT’, ‘AAT’, ‘AAAT’, ‘AAAAT’, and ‘AAAAAT’, among them ‘A’ motif showed relative abundance of 180.41 loci/Mb, thereafter ‘AAT’ (36.88 loci/Mb) and ‘AT’ (36.79 loci/Mb) motifs in *C. sinensis* (Supplementary Fig. 2A), and in case of *C. maxima*, ‘A’ motif showed relative abundance of 194.24 loci/Mb, thereafter ‘AT’ (37.77 loci/Mb) and ‘AAT’ (37.14 loci/Mb) motifs. In both the genomes, CG-rich repeats of SSRs were uncommon. Interestingly observed that, an inverse association between motif repeat number and SSR abundance, with hexa- and tetranucleotide repeats showing the strongest tendency (Supplementary Fig. 2B).

### Chromosome specific SSRs distribution

The maximum SSRs (13,680 perfect, 57,338 imperfect) were allocated to the largest chromosome 5 (36.15 Mb) while lowest number of SSRs (6712 perfect, 28,977 imperfect) were allocated to the shortest chromosome 9 (18.45 Mb) in *C. sinensis* (Table 3). In contrast, shortest chromosome 8 (21.03 Mb) in *C. maxima* having 8715 perfect, and 35,719 imperfects SSRs. While maximum 20,588 perfect SSRs were assigned to second largest chromosome 5 (49.53 Mb) and 84,595 imperfect SSRs were assigned to the largest chromosome 2 (53.00 Mb) in *C. maxima* (Table 4). As a result, chromosomal length in *C. sinensis* but not in *C. maxima* directly correlated with SSR abundance. The SSR densities on different chromosomes of *C. sinensis* and *C. maxima* were ranged from 336.71 (Chr-8) to 326.45 (Chr-9) per Mb and 378.46 (Chr-5) to 437.47 (Chr-7) per Mb with an average density of 361.69 and 398.32 SSRs per Mb, respectively (Tables 3 and 4). In the whole genome of *C. sinensis* and *C. maxima* (Supplementary Fig. 3A,B), the intra-chromosomal distribution of SSR motif types represented the frequency of mononucleotide repeats and the least presence of hexanucleotide repeats. The distribution of three major classes of perfect SSRs presented in Table 5. After excluding mononucleotide, 31,678 SSRs in *C. sinensis* and 83,605 SSRs in *C. maxima* were selected for classification. The maximum number of motifs in *C. sinensis* and in *C. maxima* (19,187; 60.57% and 64,671; 77.35%) were belong to class III, thereafter class II (10,487; 33.10% and 15,442; 18.47%) and class I (2004; 6.33% and 3492; 4.18%) across all the nine chromosomes, respectively. The overall distribution graph for three major SSR classes in each chromosome of *C. sinensis* revealed that Chr-5 and Chr-2 had the highest number of all three classes of SSRs, followed by Chr-9 that had Class I and Class III and Chr-1 consisted of Class II. However, for *C. maxima,* Chr-5 had the highest number of all three classes of SSRs followed by Chr-2 (Table 5).

**Table 3:** The chromosome-wise distribution of perfect, compound, and imperfect SSRs of *Citrus sinensis.*

Chromosome	Total Mb	Perfect								Compound		Imperfect	
		Mono	di	tri	tetra	Penta	Hexa	Total	SSRs/Mb	Total	**SSRs/Mb**	**Total**	**SSRs/Mb**
Chr-1	28.80	5265	2136	1750	878	229	100	10,358	359.64	414	14.37	45,868	1592.60
Chr-2	30.84	5735	2286	1854	903	297	138	11,213	363.62	387	12.55	49,210	1595.81
Chr-3	28.71	5637	2050	1712	866	236	106	10,607	369.40	374	13.02	45,549	1586.30
Chr-4	19.95	3646	1419	1252	600	231	62	7210	361.35	231	11.58	31,721	1589.78
Chr-5	36.15	7231	2639	2261	1054	353	142	13,680	378.46	493	13.64	57,338	1586.29
Chr-6	21.18	4123	1470	1220	580	206	85	7684	362.80	252	11.9	33,554	1584.26
Chr-7	32.21	5825	2392	1986	978	289	107	11,577	359.48	394	12.23	50,538	1569.26
Chr-8	22.71	3991	1405	1304	639	212	96	7647	336.71	250	11.01	34,590	1523.06
Chr-9	18.45	3513	1269	1121	574	185	50	6712	363.78	259	14.04	28,977	1570.51
Total	239.00	44,966	17,066	14,460	7072	2238	886	86,688	3255.25	3054	114.34	377,345	14,197.87

**Table 4:** The chromosome-wise distribution of perfect, compound, and imperfect SSRs of *Citrus maxima.*

Chromosome	Total Mb	Perfect								Compound		Imperfect	
		Mono	di	tri	tetra	Penta	Hexa	Total	SSRs/Mb	Total	SSRs/Mb	**Total**	**SSRs/Mb**
Chr-1	32.08	6804	2393	2105	972	315	130	12,719	396.46	526	16.40	53,001	1652.09
Chr-2	53.01	10,899	3812	3071	1512	518	214	20,026	377.80	768	14.49	84,595	1595.92
Chr-3	30.67	6553	2289	1949	964	291	141	12,187	397.42	517	16.86	51,422	1676.88
Chr-4	29.35	6229	2198	1940	860	286	145	11,658	397.27	449	15.30	47,472	1617.70
Chr-5	49.53	10,993	3973	3282	1593	508	239	20,588	415.67	843	17.02	82,977	1675.29
Chr-6	23.64	5552	1801	1558	697	239	125	9972	421.91	357	15.10	40,518	1714.29
Chr-7	22.27	5180	1860	1593	727	255	127	9742	437.47	372	16.70	38,278	1718.89
Chr-8	21.03	4841	1561	1357	659	206	91	8715	414.46	336	15.98	35,719	1698.68
Chr-9	40.40	7326	2361	2071	992	293	144	13,187	326.45	516	12.77	121,614	3010.58
Total	301.96	64,377	22,248	18,926	8976	2911	1356	118,794	3584.90	4684	140.63	555,596	16,360.30

**Table 5:** Distribution of three major classes of SSRs in different chromosomes of *C. sinensis* and *C. maxima.*

Chr	*C. sinensis*									*C. maxima*								
	Class I						Class II	Class III	Total	Class I						Class II	Class III	Total
	Di	Tri	Tetra	Penta	Hexa	total				Di	Tri	Tetra	Penta	Hexa	total			
Chr-1	103	70	7	3	20	203	1007	1894	3104	220	100	19	9	25	373	1563	6239	8175
Chr-2	117	93	19	4	24	257	1134	2053	3444	297	157	29	25	33	541	2463	9982	12,986
Chr-3	104	66	9	8	16	203	974	1847	3024	178	104	5	6	22	315	1530	6018	7863
Chr-4	64	42	7	3	10	126	753	1276	2155	201	82	15	11	27	336	1345	5705	7386
Chr-5	103	106	12	9	27	257	1363	2339	3959	325	148	29	16	40	558	2646	10,241	13,445
Chr-6	53	42	5	5	8	113	762	1317	2192	179	76	9	11	28	303	1199	4938	6440
Chr-7	85	74	11	7	20	197	1232	2134	3563	167	85	8	5	22	287	1288	4796	6371
Chr-8	56	46	4	6	15	127	701	1318	2146	124	69	5	8	11	217	1040	4416	5673
Chr-9	58	39	6	4	11	118	671	1208	1997	224	111	14	18	24	391	1469	6636	8496
Total	743	578	80	49	151	1601	8597	15,386	25,584	1915	932	133	109	232	3321	14,543	58,971	76,835

**Figure 1:**
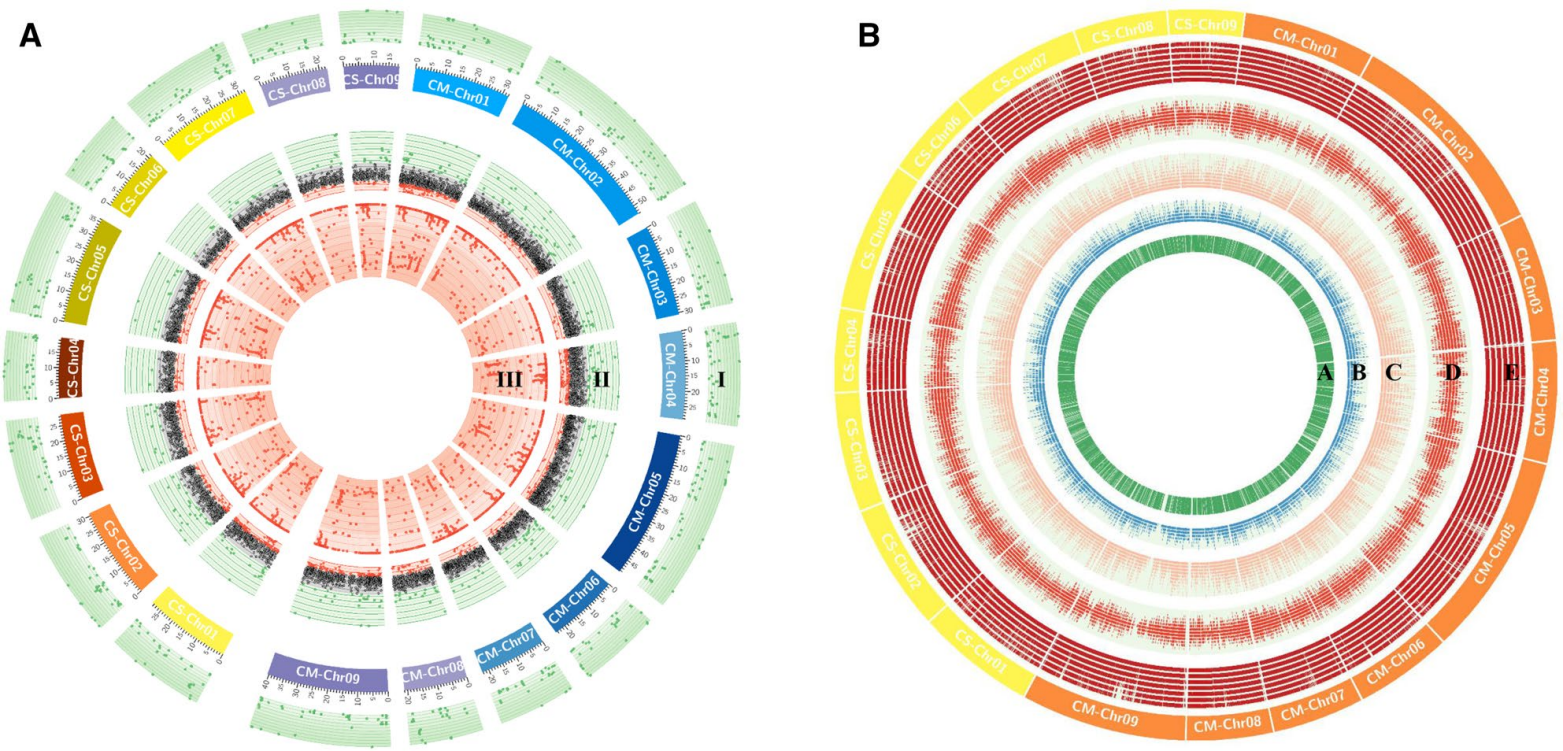
(**A**) The chromosome-wide distribution of three major groups of perfect SSRs is depicted in a Circos (mononucleotides are excluded). Class III is represented by the inner ring (III), while class II and class I are represented by the middle (II) and outer (I) rings, respectively. Abbreviation CS and CM are *Citrus sinensis* and *Citrus maxima* respectively. (**B**) The Circos feature subrings that represent (**A**) di-, (**B**) tri-, (**C**) tetra-, (**D**) penta-, and (**E**) hexanucleotides from inside to outside. Maximum variations were found in the di and tri, which can be used to create chromosome-specific hypervariable SSR markers. Abbreviation CS and CM are *Citrus sinensis* and *Citrus maxima* respectively.

Moreover, the overall distribution of class I SSRs in *C. sinensis* and *C. maxima* with respect to the number of repeat units for dinucleotides to hexanucleotides in each chromosome were studied (Table 5), and depicted in Fig. 1A,B. Circos graph (Fig. 1A) was represented all the three classes on each chromosome (III-inside, II-middle, and I-outside). Dinucleotides to hexanucleotides SSR motifs were decreased from inside to outside rings of circos graph (Fig. 1B). SSR markers from both the genomes (*C. sinensis* and *C. maxima*) were distributed intra-chromosomal basis, motifs like dinucleotides (930 and 2010) were found maximum thereafter trinucleotides (713 and 986) and the pattern were consistent in all chromosomes. According to the obtained results, the frequency of SSR markers decreased with number of repeat motifs were increased except the hexanucleotides in all the chromosomes (Table 5).

### Construction of physical map from hypervariable SSRs in citrus

Class I SSRs were used to develop primer 1602 in *C. sinensis* and 3321 in *C. maxima* on all chromosomes from both the genomes (Supplementary Table S1). The majority of primers were created for Chr-2 and Chr-5 (257) followed by Chr-1 and Chr-3 (203) in *C. sinensis* and Chr-5 (558) followed by Chr-2 (541), Chr-9 (391) in *C. maxima*, which have the highest concentrations of class I motif content. The majority of these primers were specific to *C. sinensis* and *C. maxima* to dinucleotide motifs (primers 930, 46.41% and 2010, 57.56%), followed by trinucleotide repeats (713, 35.58% and 986, 28.24%), respectively (Table 6). For experimental validation, on a set of 321 in *C. sinensis* and 1206 in *C. maxima* **extremely variable** SSRs targeting ≥ 40 nt tract length from each chromosome through ePCR on seven citrus genomes (Supplementary Table S2). The majority of SSR primers in *C. sinensis* and *C. maxima* used for validation were dinucleotides (117 and 737) or trinucleotides (158 and 383), respectively. The genomic location of 321 SSRs in *C. sinensis* and 1206 SSRs in *C. maxima* were examined (Supplementary Table S2) and represented on chromosomes (Supplementary Fig. 4A,B), of which Chr-5 (59 and 210 markers), and Chr-2 (48 and 206) had maximum number of assigned SSR markers, respectively, followed by Chr-3 (46), in *C. sinensis* and Chr-1 (145) in *C. maxima*. It is an interesting to note that scatter plots showed the physical distance (Mb), gaps between SSRs, and lengths of their tracts on each chromosome (Supplementary Fig. 4A,B). On the basis of track length all the SSR markers were ranged from 40–49nt tr (230 in *C. sinensis* and 669 in *C. maxima*), followed by 50 – 59nt (62 in *C. sinensis* and 282 in *C. maxima*), and > 70nt (87) in *C. sinensis* and (165) in *C. maxima* (Table 7). For tract length 40 – 49nt, had most markers Chr-3 (35 in *C. sinensis*) and Chr-2 (116 in *C. maxima*), whereas, Chr-4 (7 in *C. sinensis*) and Chr-8 had the least number of markers (39 in *C. maxima*). It is also interesting to note that Chr-1 had highest track length markers (204 bp), followed by Chr-2 (196 bp), Chr-3 and Chr-7 (195 bp) in *C. sinensis* (Supplementary Fig. 4A) and in *C. maxima* Chr-4 had highest track length markers (138 bp), thereafter Chr-2 (126 bp), Chr-6 (123 bp) (Supplementary Fig. 4B).

**Table 6:** Description of chromosome-specific class I SSR markers designed for nine chromosomes of *C. sinensis* and *C. maxima.*

Chr	*C. sinensis*													*C. maxima*										
	Number of class I (> 30 nt) primers						Extremely variable SSRs (≥ 40 nt) primers							Number of class I (> 30 nt) primer					Extremely variable SSRs (≥ 40 nt) primers					
	Di	Tri	Tetra	Penta	Hexa	Total	Di	Tri	Tetra	Penta	Hexa	Total	Di	Tri	Tetra	Penta	Hexa	total	Di	Tri	Tetra	Penta	Hexa	total
Chr-1	103	70	7	3	20	203	11	23	4	0	2	40	220	100	19	9	25	373	85	51	5	2	2	145
Chr-2	117	93	19	4	24	257	18	25	4	1	0	48	297	157	29	25	33	541	122	62	16	3	3	206
Chr-3	104	66	9	8	16	203	16	25	2	1	2	46	178	104	5	6	22	315	62	41	2	0	3	108
Chr-4	64	42	7	3	10	126	8	6	1	0	1	16	201	82	15	11	27	336	83	25	6	1	2	117
Chr-5	103	106	12	9	27	257	17	35	2	2	3	59	325	148	29	16	40	558	137	58	9	3	3	210
Chr-6	53	42	5	5	8	113	9	11	2	0	0	22	179	76	9	11	28	303	67	28	6	1	3	105
Chr-7	85	74	11	7	20	197	18	15	7	2	0	42	167	85	8	5	22	287	59	38	4	0	3	104
Chr-8	56	46	4	6	15	127	10	13	2	0	1	26	124	69	5	8	11	217	43	29	2	0	1	75
Chr-9	58	39	6	4	11	118	10	5	3	1	3	22	224	111	14	18	24	391	79	51	3	2	1	136
Total	743	578	80	49	151	1601	117	158	27	7	12	321	1915	932	133	109	232	3321	737	383	53	12	21	1206

**Table 7:** Classification of 1206 and 321 highly variable chromosome-specific SSR markers based on their tract lengths *C. maxima* and *C. sinensis* respectively.

Tract length (nt)	*C. sinensis*										*C. maxima*									
	Chr-1	Chr-2	Chr-3	Chr-4	Chr-5	Chr-6	Chr-7	Chr-8	Chr-9	Total	Chr-1	Chr-2	**Chr-3**	**Chr-4**	**Chr-5**	**Chr-6**	**Chr-7**	**Chr-8**	**Chr-9**	**Total**
40–49	21	29	35	7	23	13	28	12	15	230	74	116	61	60	109	54	57	39	68	669
50–59	8	7	4	3	12	1	4	6	3	62	33	33	24	21	50	28	21	22	29	282
60–69	2	4	1	2	9	1	2	1	0	28	14	30	15	21	24	11	14	10	15	163
70–162	9	8	6	4	15	7	8	7	4	87	24	27	8	15	27	12	12	4	24	165
Total	40	48	46	16	59	22	42	26	22	321	145	206	108	117	210	105	104	75	136	1206

**Figure 2:**
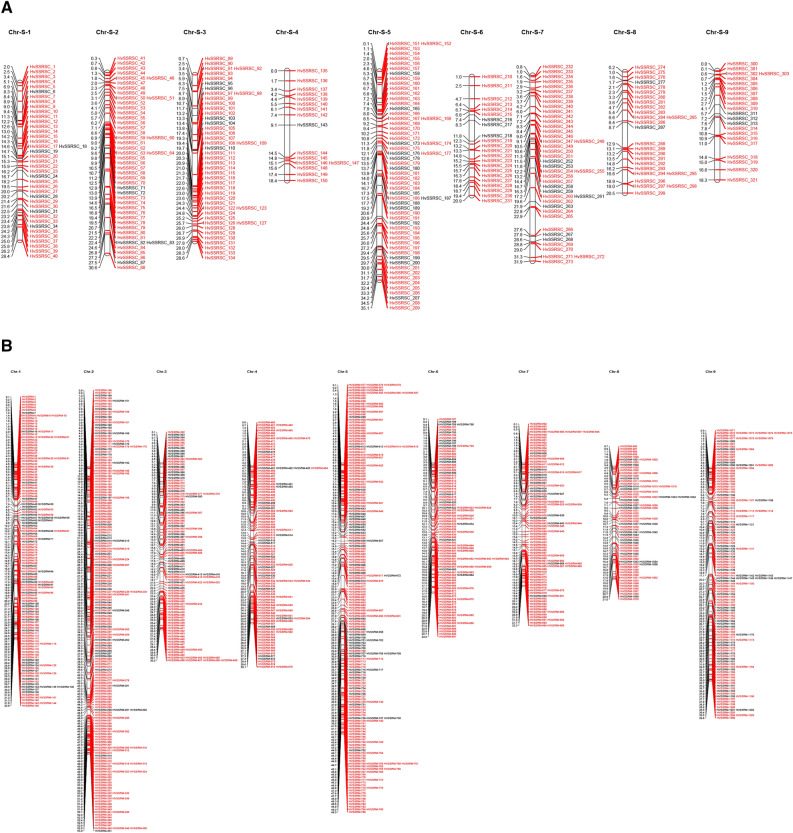
(**A**) A high-density map of 321 highly variable SSR markers (HvSSRCS) showing their physical locations on nine chromosomes of *Citrus sinensis.* (**B**) A high-density map of 1206 highly variable SSR markers (HvSSRCM) showing their physical locations on nine chromosomes of *Citrus maxima.*

The physical position and start positions of 321 HvSSRCS markers in *C. sinensis* and 1206 HvSSRCM markers in *C. maxima* on nine chromosomes were determined and these markers were used to construct a saturated physical map (Fig. 2A,B) showed that Chr-5 had the maximum number of SSR markers (59 in *C. sinensis* and 210 in *C. maxima*), followed by Chr-2 (48*C. sinensis* and 206 *C. maxima*), respectively.

### ePCR validation of the identified HvSSRs across the seven citrus species

A total of 2004 and 3492 class I SSR primers were tested on the genomes of *C. sinensis* and *C. maxima* by in silico analysis, respectively, to determine the SSRs amplification, specificity, and efficiency. The SSR markers were produced one to greater than three alleles in both genomes, and we were validated equal portions of SSR markers across the nine chromosomes (Supplementary Table S3A). A total of 1343 (83.89%) in *C. sinensis* and 2601 (78.52%) in *C. maxima* SSR markers were produced a single amplicon of designed product size, whereas 132 (8.24%) in *C. sinensis* and 211 (6.58%) in *C. maxima* primers had two alleles, and 95 (5.93%) in *C. sinensis* and 401 (11.59%) in *C. maxima* primers were produced greater than three alleles, respectively. Moreover, to validate HvSSRs with tract lengths of greater than forty nucleotides, located on physical map, in-silico PCR amplification for 321 in *C. sinensis,* and 1206 in *C. maxima* SSR markers were tested on all the available seven genome assemblies in citrus database (*C. sinensis, C. maxima, C. clementina, C. medica, C. ichangensis, Atalantia buxifolia,* and *Fortunella hindsii*).

We validated in-silico all 321 (100%) SSR markers in *C. sinensis* genome to that of 235 (73.20%), 65 (20.24%), 100 (31.15%), 107 (33.33%), 35 (10.90%), and 101 (31.46%) in *C. maxima, C. clementina, C. medica, C. ichangensis, Atalantia buxifolia,* and *Fortunella hindsii* genomes, respectively. In *C. sinensis* (272), *C. maxima* (198), *C. clementina* (54), *C. medica* (78), *C. ichangensis* (54), *Atalantia buxifolia* (30), and *Fortunella hindsii* (75) SSRs showed single-locus amplification (Supplementary Table S3A). Two hundred and twenty-one (81.25%) of these HvSSRs were polymorphic in all seven citrus genomes tested. Thereafter, 725 alleles were amplified across the all nine chromosomes. The Na varied from 2 to 6 on each locus, with a mean of 2.64 alleles. The MAF ranged between 0.58 and 1.00, with a mean of 0.74 per locus. the PIC varied from 0.80 to 0.98, with a mean of 0.75, (Supplementary Table S4). Out of 321 HvSSRCS validated, 221 SSRs showed PIC values ≥ 0.80. For the seven genomes tested, the mean Shannon information index was 0.67. The comparison of all marker parameters for each chromosome of *C. sinensis,* and *C. maxima* has been summarized in Table 8. Chr-4 had a minimum number of polymorphic SSR markers (11) and Chr-9 had higher average value of Ne (1.96), and Shannon’s information index (0.83). Furthermore, we validated 1206 (100%) SSRs in *C. maxima* genome to that of 560 (46.43%), 272 (22.55%), 394 (32.66%), 369 (30.55%), 184 (15.23%), and 168 (13.93%) in *C. sinensis, C. clementina, C. medica, C. ichangensis, Atalantia buxifolia,* and *Fortunella hindsii* genomes, respectively. In *C. maxima* (935), *C. sinensis* (2032), *C. clementina* (179),* C medica* (278), *C. ichangensis* (232), *Atalantia buxifolia* (135), and *Fortunella hindsii* (904) SSRs showed single-locus amplification (Supplementary Table S3A). We were selected a subset of 272 from *C. sinensis* and 935 from *C. maxima* to validate these chromosome-specific SSRs across seven citrus species. Thereafter calculate the marker parameters, the various amplicons were found using ePCR for these SSR markers across the seven citrus genomes (Supplementary Table S3B). Out of these, 701 (74.97%) HvSSRs were found polymorphic SSR markers across the seven citrus genomes. A total of 2139 alleles were amplified across the all the nine chromosomes. The Na values varied from 2 to 6, with a mean of 2.67 alleles per locus. The MAF ranged between 0.38 and 1.00, with a mean of 0.76 per locus. The PIC varied from 0.76 to 0.98, with a mean of 0.69 (Supplementary Table S4). 701 SSR markers showed PIC values ≥ 0.76. Out of these 935 HvSSRCM primer pairs were verified. After seven genomes analysed, the mean Shannon information index was 0.63. At the chromosomal level, we compared every marker parameter (Table 8). Chr-8 exhibited the fewest polymorphic markers (41), the highest average value of Ne (1.84), and the lowest Shannon's information index (0.68) among the nine chromosomes.

**Table 8:** Chromosome-specific marker statistics for 272 and 935 highly variable SSR primer pairs assayed through ePCR across the 7 citrus genotypes based on their genome sequences (*C. sinensis* and *C. maxima*) respectively.

*C. sinensis*												*C. maxima*										
Chr	TP	TPP	N	Na	MAF	Ne	I	Ho	He	uHe	PIC	TP	TPP	N	Na	MAF	Ne	I	Ho	He	uHe	PIC
Chr-1	33	18	2.58	2.21	0.83	1.53	0.46	0.34	0.26	0.3	0.49	102	78	2.86	2.56	0.75	1.75	0.64	0.5	0.37	0.44	0.70
Chr-2	42	37	2.95	2.88	0.71	1.90	0.77	0.58	0.44	0.52	0.82	165	123	2.65	2.56	0.76	1.74	0.64	0.49	0.36	0.44	0.69
Chr-3	41	38	2.98	2.81	0.71	1.87	0.76	0.59	0.44	0.53	0.86	79	61	2.49	2.42	0.76	1.7	0.61	0.48	0.36	0.44	0.72
Chr-4	15	11	2.47	2.27	0.77	1.65	0.56	0.46	0.34	0.41	0.68	98	70	2.7	2.6	0.76	1.74	0.63	0.48	0.35	0.42	0.66
Chr-5	45	35	2.67	2.62	0.75	1.75	0.65	0.49	0.37	0.45	0.73	166	132	2.68	2.56	0.75	1.75	0.65	0.5	0.38	0.46	0.74
Chr-6	19	13	2.32	2.26	0.78	1.64	0.55	0.43	0.32	0.39	0.65	88	63	2.46	2.39	0.77	1.68	0.59	0.46	0.34	0.41	0.67
Chr-7	34	29	2.85	2.62	0.74	1.77	0.68	0.52	0.39	0.47	0.82	90	69	3.04	2.68	0.73	1.84	0.68	0.54	0.39	0.46	0.69
Chr-8	24	22	3.13	3.08	0.70	1.93	0.81	0.6	0.45	0.54	0.85	53	41	2.47	2.4	0.76	1.69	0.6	0.47	0.35	0.43	0.72
Chr-9	19	18	3.32	3.05	0.69	1.96	0.83	0.63	0.47	0.56	0.87	94	64	2.71	2.55	0.77	1.72	0.61	0.46	0.34	0.4	0.62

TP = Total Primer; TPP = Total Polymorphic Primer; N = Average number of Alleles; Na = No. of Different Alleles; MAF = Major Allelic Frequency; Ne = No. of Effective Alleles = 1/(Sum pi^2); I = Shannon's Information Index = − 1* Sum (pi * Ln (pi)); Ho = Observed Heterozygosity = No. of Hets/N; He = Expected Heterozygosity = 1—Sum pi^2; uHe = Unbiased Expected Heterozygosity = (2N/(2N−1)) * He; Where pi is the frequency of the ith allele for the population & Sum pi^2 is the sum of the squared population allele frequencies; PIC = Polymorphic Information Content.

### Development of SSR-based physical map in citrus

High-density physical map was generated on nine chromosomes with the help physical positions of 321 HvSSRCSs in *C. sinensis* and 1206 HvSSRCMs in *C. maxima* (Supplementary Table S2, Fig. 2A,B) which showed that Chr-5 (59 and 210 markers) and Chr-2 (48 and 206) had a maximum number of allocated markers, respectively, followed by Chr-3 (46) in *C. sinensis* and Chr-1 (145) in *C. maxima* (Supplementary Fig. 4A,B). It is an interesting to note that scatter plots showed the physical distance (Mb), the intervals between SSR markers, and the lengths of each SSRs track on each chromosome (Supplementary Fig. 4A,B). Most of the primers were ranged from of 40–49 nt track length (230 in *C. sinensis* and 669 in *C. maxima*), followed by 50–59 nt (62 in *C. sinensis* and 282 in *C. maxima*), and > 70 nt (87 in *C. sinensis* and 165 in *C. maxima*) (Table 7). For tract length 40–49 nucleotide, maximum markers were located on Chr-3 (35 in *C. sinensis*) and Chr-2 (116 in *C. maxima*), whereas, Chr-4 (7 in *C. sinensis*) and Chr-8 had the least number of markers (39 in *C. maxima*). It is an interesting to observed that Chr-1 showed the maximum track length of SSR markers (204 bp), thereafter Chr-2 (196 bp), Chr-3, and Chr-7 (195 bp) in *C. sinensis* (Supplementary Fig. 1A). Similarly, Chr-4 had the maximum track length markers (138 bp), thereafter, Chr-2 (126 bp), and Chr-6 (123 bp) in *C. maxima* (Supplementary Fig. 4B).

### In vitro PCR amplification

**Figure 3:**
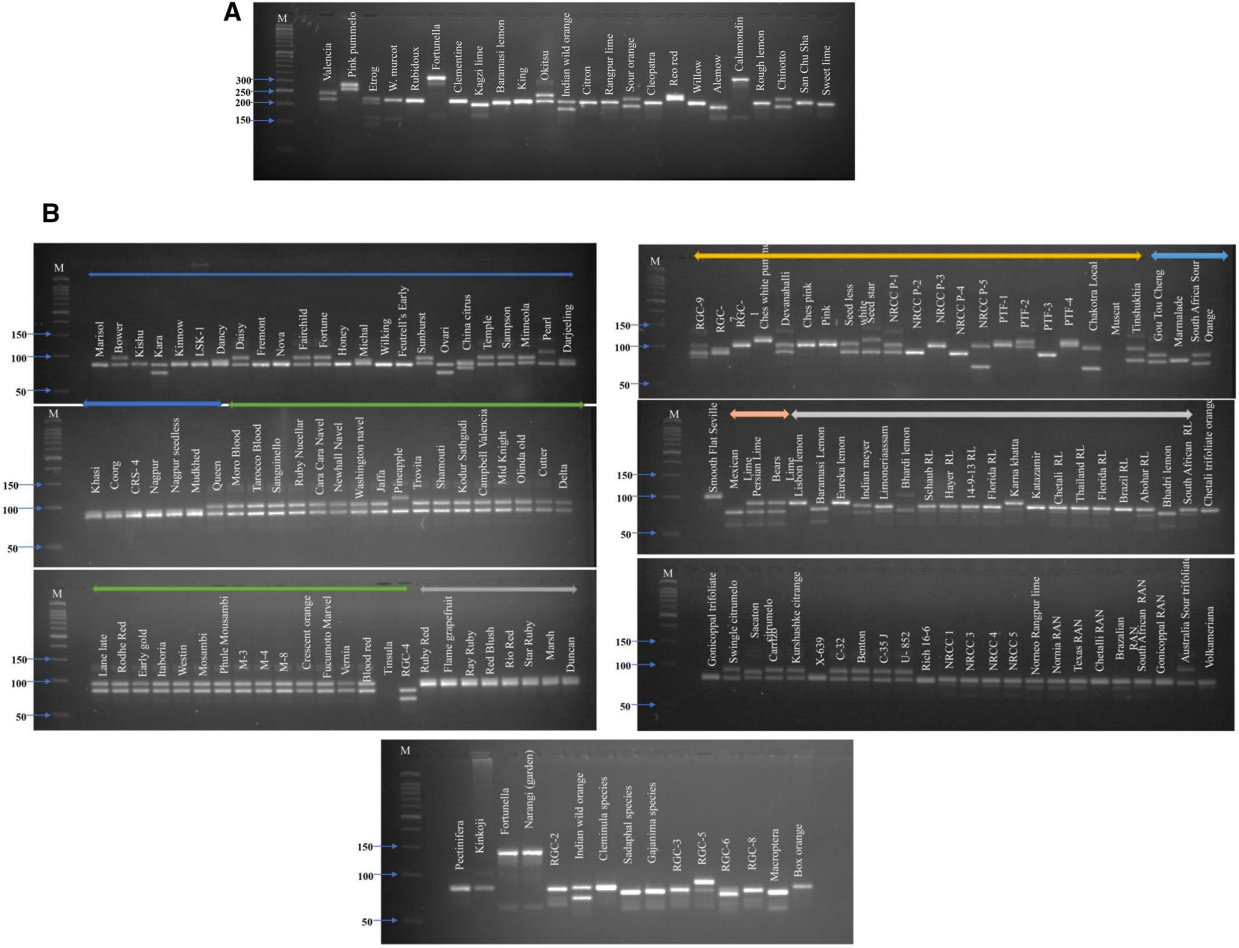
(**A**) Allelic variations revealed by HvSSRCS-116 marker when assayed on 24 citrus genotypes using **Agarose gel electrophoresis**. (**B**) Allelic variations revealed by HvSSRCS-116 markers when assayed on 159 citrus genotypes using **Agarose gel electrophoresis**.

Initially, 129 primer pairs were screened on 24 citrus species for wet-lab validation. Out of 129 primers, 2 primer pairs did not amplify **on all the 24 genotypes**, while 127 (98.45%) **primer were amplified** (Supplementary Table S5) **but some genotypes were not produced amplicons for all 127 primers.** SSR (HvSSRCS-22) profiles of 24 citrus species were depicted on gel picture (Fig. 3A,B). 786 and 693 alleles were detected using 68 HvSSRCS and 61 HvSSRCM from *C. sinensis* and *C. maxima,* respectively among the 24 citrus species (Supplementary Table S5). The number of different alleles ranged from 2 to 22 in *C. sinensis* and 4 to 22 in *C. maxima* for each locus, with an average of 12.0 in both the species. The major allelic frequency for each locus ranged from 0.13 to 0.60 and 0.13 to 0.83 with a mean 0.26 and 0.28 in *C. sinensis* and *C. maxima*, respectively. The observed heterozygosity was varied from 0 to 0.96 and 0 to 1.0 with an average of 0.51 and 0.59, and expected heterozygosity were varied from 0.58 to 0.94 and 0.29 to 0.93 with a mean of 0.84 and 0.82 in *C. sinensis* and *C. maxima*, respectively. Polymorphic information content for each locus varied from 0 (for HvSSRCS-115) to 0.95 (HvSSRCS-288) and 0.38 (for HvSSRCM-1131) to 0.92 (HvSSRCM-316) with an average of 0.80 and 0.82 in *C. sinensis* and *C. maxima*, respectively. A total of 66 HvSSR markers in *C. sinensis* and 58 HvSSR markers in *C. maxima* showed PIC values ≥ 0.5. It is interesting to observed that 49 HvSSR markers in *C. sinensis* and 45 HvSSR markers in *C. maxima* showed PIC values ≥ 0.80.

Finally, 17 HvSSRs positioned on nine chromosomes were amplified on 181 genotypes to assess genetic relationships (Supplementary Table S6). The primer sets were showed clear amplification with well-resolved fragments. The HvSSRs would be able to discriminate between the different citrus germplasm. Moreover, some HvSSR markers did not show clear discrimination among accessions of few sub-groups due to occurrence of spontaneous mutation, like in sweet oranges, grapefruits, and some mandarins. Total primer, total polymorphic primer, average number of alleles, number of different alleles, major allelic frequency, number of effective alleles, shannon's information index, observed heterozygosity, expected heterozygosity, unbiased expected heterozygosity, polymorphic information content was calculated to determine the genetic diversity/variability within whole germplasm (181) (Table 7). Based on taxonomic classifications, the overall germplasm was divided into 11 sub-groupings according to citrus variety collection (UCR: Citrus Variety Collection). The observed heterozygosity in the population as a whole was 0.69. Citrons, excluding trifoliate hybrids, have the lowest observed heterozygosity of the citrus groupings that are considered to be true Citrus species. Grapefruit was showed the maximum observed heterozygosity of all eleven taxonomic groups at 0.92.

### Cluster analysis of subsets (24) and all the citrus accessions (181)

**Figure 4:**
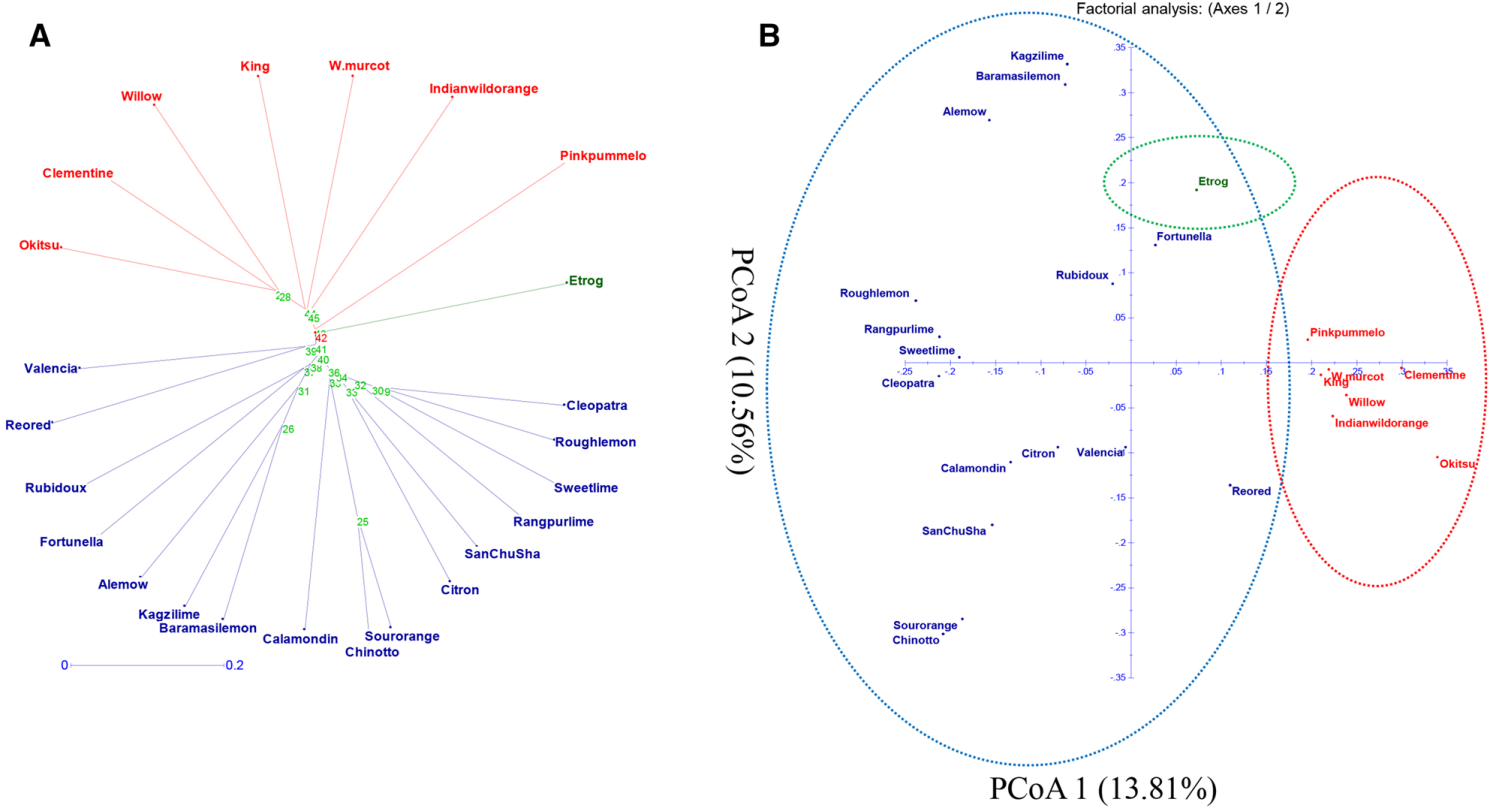
Genetic diversity among 24 citrus genotypes based on 127 HvSSR markers: (**A**) Neighbor-Joining Tree and (**B**) Principal Coordinate Analysis.

The 24 citrus species were divided into three main groups: group 1, which had 7 species, group 2, which contained 16 species, and cluster 3, which contained one citrus species (Fig. 4A). The main coordinates (PCoAs) 1 and 2 accounted 13.81% and 10.56%, respectively, 24.37% for the total variation among the 24 citrus species (Fig. 4B).

**Figure 5:**
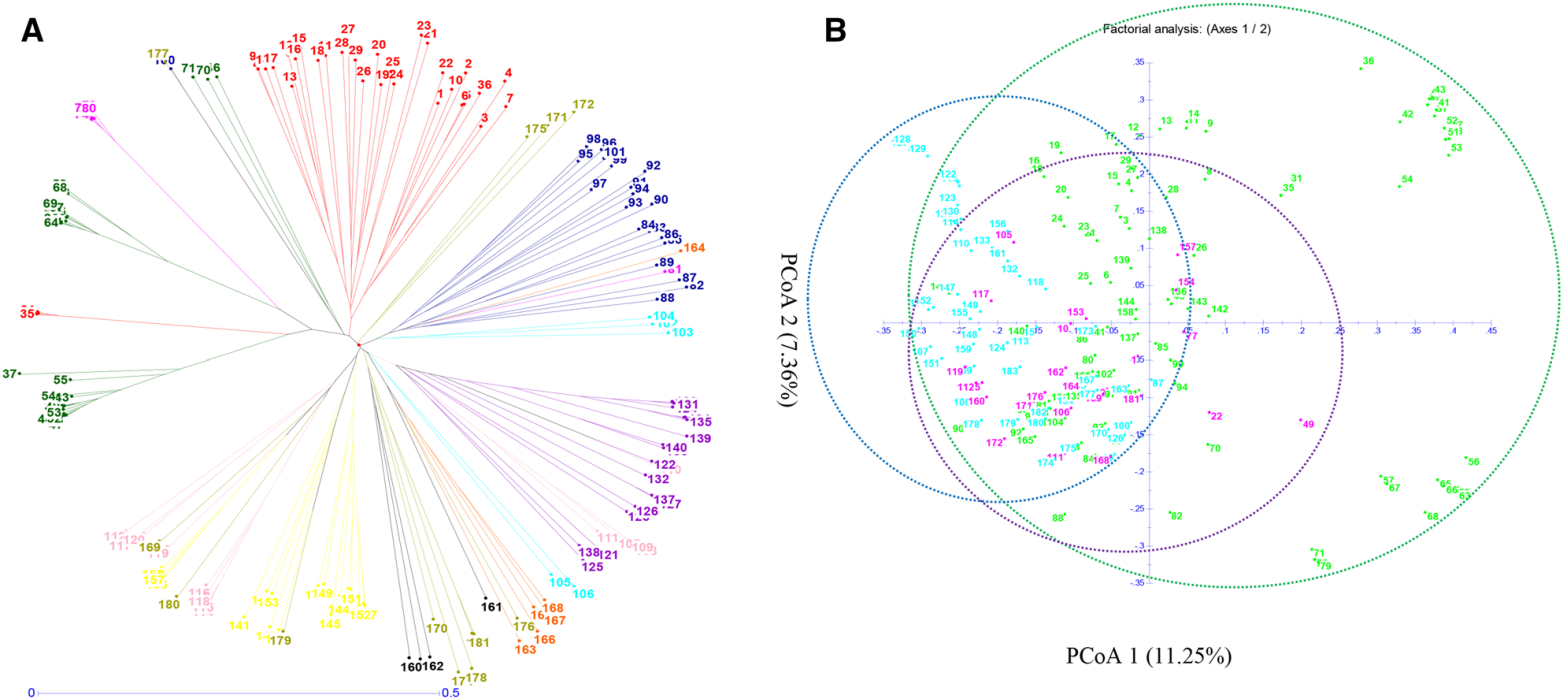
Genetic diversity among 181 citrus genotypes based on 17 HvSSR markers: (**A**) Neighbor-joining tree and (**B**) principal coordinate analysis.

In the Neighbor Joining tree, all 181 citrus accessions were clustered into three major grouped: group 1 included 24 (Pummelo and sour orange) while cluster 2, 85 (Sweet orange, Mandarin and Grapefruit) genotypes and cluster 3, 72 (Lime, Lemons, Trifoliate hybrids, Fortunella and others citrus related species) citrus genotypes (Fig. 5A). Furthermore, the PCoA allocated 181 accessions to three distinct groups (Fig. 5B). The principal coordinates (PCoA) 1 and 2 described 11.25% and 7.36%, respectively. Total variance among all the genotypes and contributed for 18.61% of the overall variation. It is an interesting to note that PCoA 1 distinguished between wild and cultivar groups for three clusters.

### Population structure

**Figure 6:**
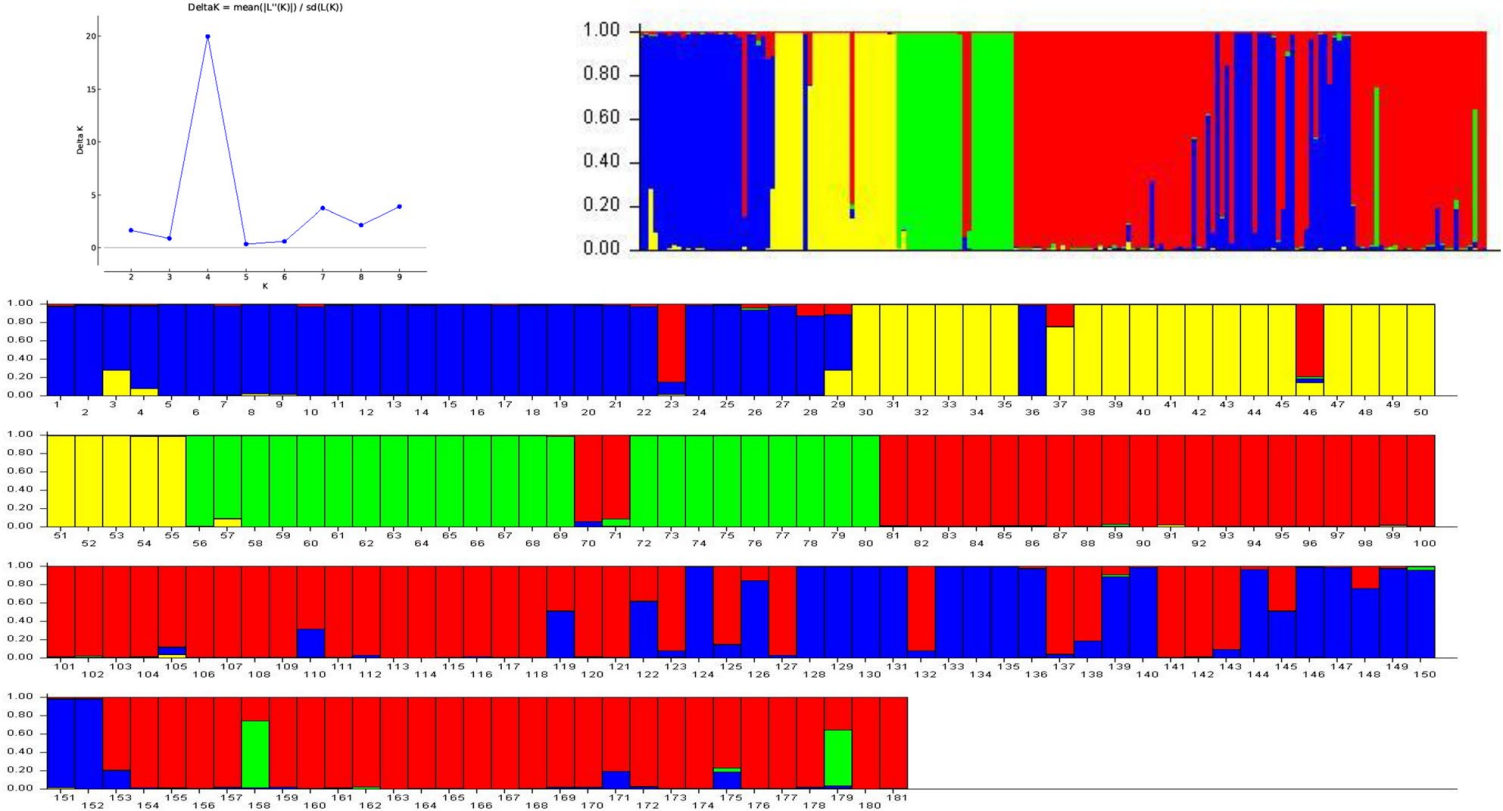
Assignment of 181 Citrus accessions by Structure v.2.3.441. Each individual bar represents an accession. Numbers 1–36 = Mandarins, 37–71 = Sweet oranges, 72–81 = Grapefruits, 82–101 = Pummelo, 102–106 = Sour oranges, 107–120 = Limes, 121–140 = Lemons, 141–158 = Trifoliate hybrids, 159–162 = Fortunella, 163–168 = Medica, 169–181 = Related species. The Y-axis displays the estimated membership of each individual in a particular cluster or population. Mandarins/lemons/Trifoliates (Blue); Mandarins/sweet orange (Yellow); sweet orange/Grapefruit (Green); pummelos, citrons, trifoliates, Sour orange, Limes and a kumquat/papeda group (Red).

Seventeen HvSSRs were employed to estimate population structure among the 181 diverse citrus genotypes (Fig. 6). These genotypes were grouped into four set: mandarins/lemons/trifoliates (blue); mandarins/sweet orange (yellow); sweet orange/grapefruit (green); pummelos, citrons, trifoliates, sour orange, limes and a kumquat/papeda group (red). Mandarins and pummelos are true citrus species, whereas, citrons, *Fortunella* and trifoliate hybrids are not classified as separate but they are related genera. The other citrus species showed mixing between two or more of these four populations, which were evident hybrids between naturally existing types (Fig. 6). An intriguing outcome of this research is that mandarins (#30–35) were segregated from other mandarins, possibly as a result of their varied geographic origins, as seen in Fig. 6. According to this research, sweet oranges (#37–45 and 47–55) have a genetic makeup that is mostly derived from mandarin and very little from pummelo, whereas #46, 70, and 71 have genetic compositions that are mostly derived from pummelo and some part from mandarin.

### GO classification of genic SSRs

The potential functions of SSR-loci were assigned using BLASTX. This method was showed that 95% of all SSR loci had no substantial resemblance to known protein-coding sequences, whereas 5% of all SSR marker had functional protein-coding sequences annotated in the public non-redundant protein database. A significant gene annotation was found in 104 loci for *C. sinensis* and 387 loci for *C. maxima*. The majority of SSR loci with an annotation were discovered to be engaged in biological activities, including oxidation reduction (27%) metabolic processes (15%), and carbohydrate metabolism (6%) in *C. sinensis*. In *C. maxima*, metabolic processes (38%) were observed, as well as biosynthetic processes (15%), reactions to stress (15%), and metabolic processes (15%) in *C. sinensis*. Among the many molecular process categories, *C. maxima* was shown to have better ATP and protein binding activity than *C. sinensis*. However, it was shown that both citrus species had a comparable 10% protein kinase activity level. **The appearance of above mention category is differed from the whole gene set in both *C. sinensis* or *C. maxima*** (Supplementary Fig. 5A,B). Similar percentages of oxidoreductase, cellular and catalytic activity were found in both species (Supplementary Fig. 5A,B).

## Discussion

HvSSR markers have been extensively used for genomic study, linkage/trait/QTL mapping, DNA fingerprinting, gene tagging, population genetics, conservation biology, and idiotype/molecular breeding in citrus breeding. However, the limited availability of chromosome specific highly variable SSR markers has impeded trait identification and mapping in citrus species because of high cross-compatibility. Kijas et al.^46^ was developed the first citrus SSR markers to improve citrus. The draft genome sequences of various citrus species, viz*., C. sinensis*, *C. clementina*, *P. trifoliata*, and *C. limon*, were used in numerous projects from 2006 to 2020 to identify a considerable number of genome-wide SSR markers^9,24,47–54^. Applicability of these markers and their efficiency for genetic mapping, genetic diversity, and population structure were investigated in additional citrus species. Additionally, Biswas et al.^24^ reported a success rate of 56.21%, Biswas et al.^9^ 65.0%, and Barkley et al.^37^ 62.50% for the PCR amplification of SSR primers in citrus. In our study success rate was much higher (98.45%) as compared to the prior studies. However, because to the lack of chromosome specific profiling of SSRs and their immediate utility in genetic investigations is severely limited.

### SSR density and their distribution in *C. sinensis* and *C. maxima* genomes

The goal of the current study was to provide SSR markers that are extremely informative chromosome-wise in citrus. The genome sequence covering perfect HvSSRs reported for 0.70% (2.31 Mb) in *C. sinensis* and 0.67% (2.31 Mb) in *C. maxima* of entire assembled genomes, which is almost similar to observed in the grapevine genome (0.67%)^55^ but lower than in pomegranate 1.74%^17^ and in eggplant 1.25%^26^. 3833 (3.52%) and 5042 (3.90%) compound type SSRs were found in the *C. sinensis* and *C. maxima* genomes, respectively, which is less than the 15,483 (8.92%) SSRs were found in the Dabenzi genome^36^ eggplant inbred line 67/3 genome was revealed 20,670 (15.6%)^26^, while Tunisia genome was showed 55,836 (15.28%)^17^. In our study, SSR density (331.86 SSRs/Mb) in *C. sinensis* and (374.00 SSRs/Mb) in *C. maxima* genome which is contrast to Morgante et al.^56^, reported that species possessing larger genomes were showed lower SSR density (SSRs/Mb). Biswas et al.^24^, were reported SSR marker density (146.42 SSR/Mb) in sweet orange cv. Valencia genome, which is 2.3-fold lower than our study. Patil et al.^17^, were reported SSR density (1,230.6 SSRs/Mb) in Tunisia genome and (387 SSRs/Mb) in jujube genome^32^ followed by peach (219 SSRs/Mb), **plum (*Prunus mume*)** (211 SSRs/Mb), and mulberry (281 SSRs/Mb). The density of SSRs has been found to be unrelated to genome size, despite the possibility that variations in genome size may influence the degree of microsatellite repetition, it may be due to track length of SSRs^55,57,58^.

According to the size of the chromosomes in *C. maxima*, the number of SSR markers and SSR densities differ. Similar with our results in *C. sinensis*, Patil et al.^17^ was observed maximum number of SSR markers (60,708 perfect SSRs, 67,141 imperfect SSRs) on longest chr-1 (55.56 Mb) and less perfect (36,241) as well as imperfect SSRs (41,901) were assigned to shorter chromosome 8 (27.99 Mb) in pomegranate, respectively. Constant observations were found in globe artichoke^55^, eggplant^26^. Regarding to intra-chromosomal distribution of SSR motifs in *C. sinensis* and *C. maxima* mononucleotide was found to be abundant SSR type thereafter dinucleotides, which is the best conformation with the prior findings^24^.

Class III SSRs were found to be the most prevalent, thereafter class II and I, when the distribution of SSR types across chromosomes were examined. The frequency of SSRs and the number of repeats on each chromosome were correlated, and these results were consistent with observations from other plant species, i.e., pepper^59^, globe artichoke^55^, eggplant^26^ and pomegranate^17^. Additionally, class I SSRs in each chromosome in both citrus species had mononucleotide dominance followed by dinucleotide repetitions. These trends were also seen in the distribution of the three main classes of SSRs over the whole genome. In contrast, Biswas et al.^9^, was reported that varied nucleotide repeats in class I as compare to our study, while class II repeats were similar to both the citrus species.

### Chromosome-specific hypervariable SSR marker—design and distribution

Class I SSR markers were used to generate primers in *C. sinensis* and *C. maxima*, 2004 and 3492 primers were specific to Chr-5 (257) and Chr-5 (558), while Chr-6 (113) add Chr-8 (217) had lower number of primers, respectively. The number of SSRs and chromosomal length in eggplant and pomegranate, respectively, were shown to be correlated, according to Portis et al.^26^ and Patil et al.^17^. In our study same results were observed in *C. sinensis* and contrast results in *C. maxima* were observed, there is no correlation in *C. maxima* between chromosome length and number of SSR markers. The present study found that, at the whole genome level, the distribution of markers for each chromosome reduces as track length increases. Similarly, Patil et al.^17^ observed that number of SSR markers decrease when track length increases in pomegranate and Portis et al.^26^ in eggplant genome. As shown across the whole genome of *C. sinensis* and *C. maxima*, mononucleotides (A and T) predominately found in each chromosome in citrus, followed by dinucleotides (AT). In contrast, Portis et al.^26^ and Patil et al.^17^ reported distribution of dinucleotide predominated followed by trinucleotide in eggplant and pomegranate genome within individual chromosomes, respectively.

### Development of high-density physical map using HvSSRs

A high-density and high-resolution genetic linkage map with consistent genomic locations and maximum coverage is essential for the mapping of genes and QTLs, which is easily achieved by recent developments in sequencing and genotyping technology^60,61^. In citrus, there are currently not enough reports on the creation of HvSSR-based physical maps. Here, using 321 and 1206 HvSSR markers, we were constructed a saturated physical map of *C. sinensis* and *C. maxima*, respectively. In both citrus species, Chr-2 had the second-highest number of markers, followed by Chr-5. This demonstrated a relationship between the number of markers and chromosomal length. With a little divergence in the sparse distribution of markers towards the middle of chromosomes as opposed to distal ends, the SSR distribution pattern revealed that each chromosome had approximately equal amounts of markers present. A highly saturated physical map may be used as a reference map for genotyping data analysis for various breeding populations and genotypes, speeding up the mapping and breeding of distinct citrus traits. Zhao et al.^62^, were reported that the utility of HvSSR markers situated on physical map and it will be applicable for fine mapping from the reported QTLs and many other crops used to estimate synteny and collinearity^17,63,64^. In consideration of this, we can predict that the data obtained here will surely be useful to citrus scientists for citrus breeding.

### Selection of single-locus SSRs through ePCR

To measure the amounts of SSR polymorphism among seven distinct citrus genome sequences, we were used an in silico-simulated PCR. In pomegranate, the ePCR approach has been used for confirmation through in-silico of molecular markers^17^. The present investigation was provided, 321 and 1206 (> 40 bp) ePCR validated SSR markers across nine chromosomes of *C. sinensis* and *C. maxima*, respectively. Among these, 272 and 935 SSR loci had single ePCR amplified product in *C. sinensis* and *C. maxima* genomes, respectively. Out of these, 272 primer pairs, in *C. maxima* (198), *C. clementina* (54), *C. medica* (78), *C. ichangensis* (54), *Atalantia buxifolia* (30), and *Fortunella hindsii* (75) via e-mapping with 81.25% polymorphic and its mean PIC value 0.75. Similarly, 935 primer pairs, in *C. sinensis* (2032)*, C. clementina* (179), *C. medica* (278), *C. ichangensis* (232), *Atalantia buxifolia* (135), and *Fortunella hindsii* (904) via e-mapping with 74.97% polymorphic and its mean PIC value 0.69. The 400 SSRs discovered from the draught genome sequence of *C. sinensis* in a prior work were showed an average PIC value of 0.73^24^, and 46 HvSSRs had PIC values 0.4 in pomegranate^36^. SSR polymorphism and track length were found to be directly proportional^13,65^ and our results were similar to these findings.

### Wet-lab validation of HvSSRs on a core set of citrus genotypes (24)

Wet-lab confirmation were done on 24 citrus accessions by 129 HvSSRs with 98.45% polymorphism recorded and these primers developed through ePCR. Novelli et al.^50^, designed SSR markers from a genome of sweet orange cv Pera IAC and found 66.08% functional SSRs. Biswas et al.^9^, synthesized SSRs from *C. clementina* BAC-end sequences (BES) reported that 83.25% amplification of SSRs and 65.00% revealed cross-species transferability with Citrus and Citrus relative species. Biswas et al.^24^ was identified 56.21% polymorphic HvSSR markers that were developed from *C. sinensis* genome. The PIC value ranged was from 0 to 0.95 with an average of 0.81 observed in our study, which is quite more than previously reported by Ravishankar et al.^66^ from twelve pomegranate genotype (0–0.91) and Patil et al.^36^ was given from 0.12 to 0.63. The use of various genotyping platforms, such as agarose, polymer gels, and automated capillary-based techniques, might be contributed to the variations in SSR allele count and PIC values seen in different studies. Mandarin and their hybrids such as Okistu, clementine^67^, king^68^, W. murcot, Indian wild orange^37^, Valencia, and pink pummelo clustered together in the same group. Citrus related species, Reo red, willow, Rubidoux, fortunella^37^, alemow, kazi lime, baramasi lemon, calamondin^37^, chinnoto, sour orange, citron, san chu sha, rangpur lime, sweet lime, rough lemon, and cleopatra species in a single cluster. One of the true citrus species *C. madica* (etrog)^37^ fall into the third cluster.

### Genetic diversity analysis of 181 citrus germplasm

The genetic diversity assessment of 181 different citrus germplasm with 17 SSRs demonstrated the usefulness of the novel HvSSRs for citrus genetic improvement. Our current findings revealed that among 181 citrus genotypes, there is a significant amount of genetic variation. Due to spontaneous mutation within some group cultivars which leads to discovered few molecular polymorphisms among them^37,69,70^. Therefore, these 17 HvSSR markers were unable to discriminate some clonally produced varieties. According to Barkley et al.^37^, the lowest reported heterozygosity was seen in citrons excluding trifoliate hybrids, which validates our findings but we observed 2.25-fold change increase in our findings. Grapefruit are apparent hybrids of pummelo and sweet orange^68^, across all taxonomic groupings, had the greatest detected heterozygosity, increasing the 1.64-fold chain. In comparison to the groups categorised as citrus ancestral or relatives, several of the groupings assumed to be hybrids of the naturally existing types of citrus showed a larger share of heterozygous loci. Limes are reportedly tri-hybrids of *Citrus medica* (citron), *Citrus maxima* (pummelo), and Microcitrus^71^ or apparent hybrids of citrons and papedas as a maternal parent^68,72^, showed the maximum observed heterozygosity of all the eleven systematic groups at 0.66, which is almost identical to Barkley et al.^37^. The sweet oranges, on the other hand, have long been believed to be a back cross between a pummelo and a mandarin (1:3 ratio)^68^, showed minimum heterozygosity (0.65) among the natural and developed hybrid sets.

Among the ancestor species, the pummelo had the greatest frequency of heterozygotes (0.74), which increased from previous research by 1.77-fold chain. Of all the taxonomic groupings, limes had the greatest observed heterozygosity (0.66). However, among the hybrid groups including sweet oranges exhibited one of the lowest heterozygosity (0.65). In the last cluster, there are six admixture groups. The citrons, kumquats, trifoliate hybrids, and species related to citrus were all grouped together with the lemon, lime, and their hybrids. Lemons are believed to be natural crossbreeds between citrons and limes or between citrons and sour oranges^39,68,73,74^. Mandarin-lime was the initial classification for the rangpurs. Rangpurs are belongs to *C. reticulata* introgressed with a few genes from *C. medica*^37,39,68^.

### Phylogenetic analysis of all accessions

In the present study, 181 diverse citrus accessions were used for phylogenetic analysis. Pummelo and pummelo hybrids, sour oranges, and a few sour orange hybrids make up the first major category. According to Scora^75^, the pummelo is regarded as a true citrus species which was used for hybridization to produced bitter grapefruits and oranges^71,75^. The pummelos were quite similar to one another because they grouped together and had very small branch lengths between accessions.

The mandarins, sweet oranges, and grapefruits made up the next significant group but did not form a well-defined clade. The delicious orange and mandarin groupings dispersed into several smaller clusters. When hybrid and nonhybrid accessions were analysed, Federici et al.^76^ discovered that *C. reticulata* group did not constitute a coherent cluster. Mandarins formed a distinct monophyletic group; hybrids were eliminated from the genotypic data (Fig. 5). *C. reticulata* is regarded as a legitimate citrus species. *C. sinensis*, assumed to a natural hybrid and majority of its genome inherited from *C. reticulata* supposed to be female parent because chloroplast genome recovered from mandarin and minute segment of genome from *C. maxima* features, is an interesting outcome of earlier reported investigation^71,72,75,77,78^. But among the hybrid varieties, the sweet oranges, which were previously believed to be a back cross of mandarin^68^, exhibited the lowest heterozygosity (0.65).

Pummelo and sweet orange were thought to be the parents of grapefruit^71,72^. This research included DNA markers based on InDel-SSR markers^79^, SNP markers^74^, and DNA fingerprinting analysis^73^. Grapefruit is a hybrid of *Citrus maxima* cross with *Citrus sinensis* were confirmed by using whole genome sequencing^48,68,80,81^. Hybrids were developed in past era through natural and man-made crossing events between *C. sinensis* (oranges), *C. reticulata* (admixed mandarins), and *C. paradisi* (grapefruits). Tangors were developed from *C. reticulata* (mandarins) and *C. sinensis* (sweet oranges), Tangelos from *C. paradisi* (grapefruit) and *C. reticulata* (mandarin), and orangelos from *C. sinensis* (sweet orange) and *C. paradisi* (grapefruit). These hybrids were considered as a small citrus variety^81^. Dendrogram showed that Fortunella and *Alalantia buxifolia* are not far from accessions in the genus *Citrus*.

### Structure analysis

The connections between citrus species and the origins of their hybrids have been better understood from the result of structure analysis of the HvSSRs data. On the other hand, the findings support one another to offer a decent analysis. The neighbour-joining tree is a distance-based approach that determines percentage of common alleles across species and then plots these distance correlations as a tree. Structure seeks to identify population structure in which each population is in linkage equilibrium and Hardy–Weinberg equilibrium. It does this by using a Bayesian clustering technique to probabilistically assign people to populations based on their genotypes.

The 181 accessions, population structure was examined using structure. If an individual genotype suggests mixing, they are allocated to a population or many populations. The majority of genetic marker systems may be used in this technique to estimate population structure, given that the markers are not strongly connected^7,37,39,68^. It makes no assumptions about the specific mutation process^37^. According to Scora^75^ and Barrett and Rhodes^71^, there are just a few naturally occurring varieties of citrus (citron, pummelo, and mandarin). These studies also give more evidence for the ancestry of the majority of other citrus species, which are thought to be hybrids descended from these species. The trifoliate hybrids, kumquats, and citrons did not cluster as a separate population despite several runs of the study. This could be as a result of the small number of genotypes included in the genotypic data and the substantial mixing that most of them exhibit. Finally, it is probable that additional molecular markers will be required to distinguish between a distinct population of trifoliate hybrids, citrons, and kumquats.

### Gene annotation

This is to be expected as the majority of SSRs are present in the intergenic regions of both the genome (*C. sinensis* and *C. maxima*). However, only 9% of the SSRs were showed notable Gene Ontology (GO) hits. SSR loci that include GO keywords which provides an excellent candidate for use as DNA markers in association analysis^24,82^. Functionally, defined SSR markers may make it easier to choose potential gene-based markers for the validation of the functional annotation and for establishing relationships between marker-phenotype associations. For trait association analysis, marker-assisted selection, building transcript base maps, comparative mapping, and evolutionary research all taken together, functional markers may offer benefits over anonymous markers^24,82^.

## Conclusion

New molecular breeding techniques aim to overcome conventional breeding limits for citrus species, in order to obtain new varieties with improved horticultural traits and resistance to biotic and abiotic stress. Earlier in citrus, two classes of SSRs were identified on the basis of track length maximum 20 nt but in this study, we described SSRs that represent nine chromosomes from *C. sinensis* and *C. maxima* genome, and increased the track length > 40 nt (**extremely variable** SSRs 321 from *C. sinensis* and 1206 *C. maxima*) because polymorphism will be increase with increase the track length. *C. sinensis* and *C. maxima* yielded a total of 1,08,833 and 1,29,321 perfect SSRs, respectively.

Through ePCR, we first evaluated the in-silico amplification of 321 HvSSRs from *C. sinensis* and 1206 HvSSRs from *C. maxima*, and we discovered 272 SSRs in *C. sinensis* and 935 in *C. maxima* that amplify a single locus in each species. Seven citrus genome assemblies were subjected to the ePCR method, which revealed 221 *C. sinensis* and 701 *C. maxima* SSRs to be polymorphic. 129 HvSSRs were validated through wet-lab and found 98.45% polymorphism. 181 genotypes were divided into 11 main groups through 17 HvSSRs. However, the genotypes were genetically dissimilar due to genetic admixture. In general, all SSR loci used in this study showed high levels of polymorphism (mean 98.45%), which were confirmed the high genetic diversity of citrus in different genotypes. The diverse genotypes of present study may be selected for cross breeding and development of mapping population in citrus breeding program for horticultural traits and resistance to biotic and abiotic stress.

## Materials and methods

The present study was conducted at Punjab Agricultural University (PAU), Ludhiana, India during the years from 2020 to 2022 with relevant institutional guidelines and legislation. Necessary permission was obtained from the institute for the collection of plant material.

### Genomic data collection

**Figure 7:**
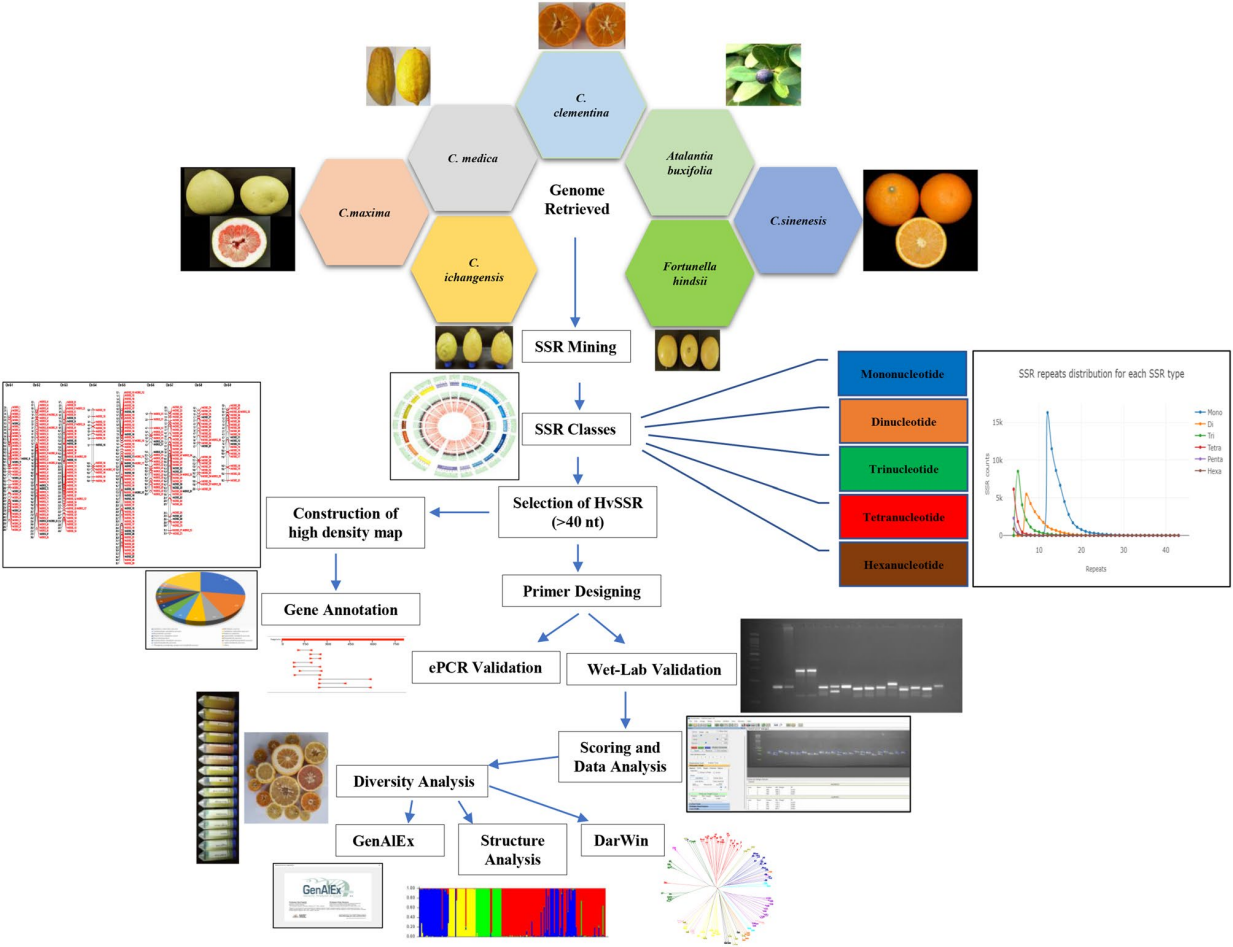
The schematic workflow of In-silico development of highly variable SSRs and its validation.

High-quality genome assemblies of *C. sinensis*^25^, and *C. maxima*^45^ were retrieved in FASTA format from the Citrus Genome Database (https://www.citrusgenomedb.org/). Electronic polymerase chain reaction (ePCR) was performed using five other draft genomes of genus citrus viz., *C. clementina, C. medica, C. ichangensis, Atalantia buxifolia,* and *Fortunella hindsii*^45,83–85^, which were retrieved and validated for identified SSR markers (Fig. 7).

### Genome-wide survey for SSR motifs and primer design

The total genome size of *C. sinensis* and *C. maxima* was 327.94 Mb and 345.78 Mb, respectively. Genome-wide sequences were surveyed for SSR mining and identification of chromosome specific perfect, compound, and imperfect SSR markers through Krait: ultra-fast SSR search module^86^ (https://github.com/lmdu/krait). The genomes of both citrus species, 2 to 6 nucleotide pattern was chosen, and the minimum repeat unit was determined as twelve for mononucleotides, seven for dinucleotides, five for trinucleotides, and four for tetranucleotides, pentanucleotides, and hexanucleotides. Two SSRs were separated by 100 bases to form compound microsatellites.

From the overall identified SSR motifs of *C. sinensis* and *C. maxima*, the chromosome specific hyper-variable SSR primers (> 30nt) were discovered. The primer3-py project (https://github.com/libnano/primer3-py), which is implemented in Krait software, was used to design the primers. Different parameters were used to design primers having amplicon size of 100–400 bp in length, primer length (nt) 18–20 (optimum 19 nt); GC content 40–70%; Tm 52–60 ^◦^C (optimum 55 ^◦^C). The other parameters were used as the default for primer designing. Genome-wide hypervariable class I SSR markers from *C. sinensis* and *C. maxima* were designated as “*C. sinensis*” (HvSSRCS) and “*C. maxima*” (HvSSRCM), respectively. A total of 272 as hypervariable SSR markers “*C. sinensis*” (HvSSRCS) and 935 as hypervariable SSR markers “*C. maxima*” were selected for ePCR.

### In silico evaluation of designed SSRs markers

The Genome-wide microsatellite analyzing tool package (GMATA) software^87^ was utilized to execute an in-silico ePCR amplification^88^ to evaluate the amplification efficiency of newly generated SSRs (class I, > 30 nt) and to map the proposed marker to genomic sequences of nine chromosomes of *C. sinensis* and *C. maxima*. The settings for ePCR were margin 3,000, no gap in primer sequence, no mismatch in primer sequence, the amplicon size range of 100–1,000, word size (-w) 12, and contiguous word (-f) 1.

The marker mapping information was processed using the ePCR results. The output file (.emap) contained information about the markers amplification patterns, such as amplicon sizes, physical chromosomal positions, as well as the unique and multiple loci mapped markers. Subsequently, **extremely variable** SSRs (class I, > 40 nt) were tested on nine chromosomes of ‘*C. sinensis* and *C. maxima*’ to identify SSRs producing one amplicon. Finally, all the identified single-locus SSR primers of *C. sinensis* and *C. maxima* chromosomes were evaluated across the five (*C. clementina, C. medica, C. ichangensis, Atalantia buxifolia,* and *Fortunella hindsii*) draft genome sequences of citrus species along with *C. sinensis* and *C. maxima*.

The produced amplicon sizes obtained for highly variable SSRs across the seven citrus genomes using GMATA were used to estimate various SSR marker parameters viz., total primer (TP); total polymorphic primer (TPP), average number of alleles (N), No. of different alleles (Na), major allelic frequency (MAF), No. of effective alleles (Ne), shannon's information index (I), observed heterozygosity (Ho), expected heterozygosity (He), unbiased expected heterozygosity (uHe), polymorphic information content (PIC) by using GenAlEx v. 6.5 software^89^.

### Construction of a highly saturated SSR-based physical map

The start and end positions of all SSR loci on each chromosome of both species, as well as their major classes, viz., classes I, II, and III, were obtained through Krait software. Circos software (http://www.circos.ca) was used to create a circular graph to show the chromosome wise distribution of different SSR markers^90^. The chromosome wise scatter plots were created through Microsoft Excel depends upon physical positions, and tract length of the hypervariable SSR markers (class I, > 40 kb) and by using MapChart v 2.2 software^91^, the physical locations of hypervariable SSRs were used to show the high density SSR based physical map of every chromosome from both citrus species.

### Experimental validation of SSR markers

181 diverse citrus germplasms were utilized to validate newly designed HvSSRs (Table 9). Plants were grown in the orchard of the Department of Fruit Science in Punjab Agricultural University, Ludhiana, India. The modified CTAB^92^ procedure was used to extract genomic DNA from healthy leaf samples of all citrus accessions (Table 9). The extracted DNA was quantified on 0.8% agarose gel electrophoresis and Thermo scientific NanoDropTM 1000 spectrophotometer and normalized to 30 ng/µl for polymerase chain reaction. For wet-lab validation, a total of 129 (68 from *C. sinensis* and 61 from *C. maxima*) chromosome wise hyper variable HvSSRCS and HvSSRCM primer were synthesized, and firstly, screened on a subset of 24 citrus germplasm (24 genotypes denoted the most of the citrus species and closely relative genera from 181 accessions) for the PCR amplificatipn and transferability analysis, Table 9 with* marks. Subsequently, 2 markers from each chromosome were selected randomly from both the species for genetic diversity analysis in 181 citrus accessions.

**Table 9:** Total germplasm used in this study.

S. no.	Common name	Parentage	Scientific name	S. No.	Common name	Parentage	Scientific name
1	Clementina Type 3*	Mandarin × Sour orange	*C. clementina* Hort.ex Tanaka	24	China citrus		
2	W. Murcott Type 3*	Seedling selection of Murcott		25	Temple	Natural selection	*C. reticulata* Blanco
3	Marisol	Mutation of 'Oroval' clementine	*C. clementina* hort. ex Tanaka	26	Sampson (Tangelo)	Grapefruit × Dancy tangerine	
4	Bower	Clementine mandarin × Orlando tangelo		27	Minneola (Tangelo)	Duncan grapefruit × Dancy mandarin	*C. reticulata* Blanco
5	King Type 3*	Natural tangor	*C. nobilis* Lour	28	Pearl (Tangelo)	Imperial grapefruit × Willow leaf mandarin	
6	Kishu small tangerine		*C. kinokuni* Hort. ex. Tanaka mukakukishu	29	Darjeeling mandarin	Natural selection	*C. reticulata* Blanco
7	Kara	King tangor × Ovari satsuma		30	Khasi mandarin	Natural selection	*C. reticulata* Blanco
8	Kinnow	*C. nobilis* Lour × *C. deliciosa* Tenora	*C. reticulata* Blanco	31	Coorg mandarin	Natural selection	*C. reticulata* Blanco
9	PAU Kinnow-1	(Induced low seeded mutant of Kinnow)	*C. reticulata* Blanco	32	CRS- 4 mandarin	(Selection from Coorg mandarin)	*C. reticulata* Blanco
17	Honey mandarin	*C. nobilis* Lour × *C. deliciosa* Tenora		33	Nagpur mandarin	Natural selection	*C. reticulata* Blanco
19	Wilking Type 3	King × Willow leaf		34	Nagpur seedless mandarin	(Seedless selection from Nagpur mandarin)	*C. reticulata* Blanco
10	Willow*		*C. deliciosa* Tenora	35	Mudkhed mandarin	(Low seeded selection from Nagpur mandarin)	*C. reticulata* Blanco
11	Dancy tangerine	Chance seedling, Natural selection	*C. reticulata* Blanco, *C. tangerina* Tanaka	36	Queen		*C. sinensis* (L.) Osbec kunshiu
12	Daisy tangerine	Fortune × Fremont mandarin		37	Moro Blood		*C. sinensis* (L.) Osbeck
13	Fremont mandarin	Clementina × Ponkan Mandarin	*C. reticulata* Blanco	38	Tarocco Blood orange		*C. sinensis* (L.) Osbeck
14	Nova mandarin		*C. reticulata* Blanco	39	Sanguinello Blood orange		*C. sinensis* (L.) Osbeck
15	Fairchild	Clementine mandarin × Orlando tangelo	*C. reticulata* Blanco	40	Ruby Nucellar (RN)		*C. sinensis* (L.) Osbeck
16	Fortune	Clementine mandarin × Orlando tangelo	*C. reticulata* Blanco	41	Cara Cara Navel		*C. sinensis* (L.) Osbeck
18	Michal	Natural hybrid of Clementina × Dancy		42	Newhall Navel		*C. sinensis* (L.) Osbeck
20	Feutrell’s Early	Clementine mandarin × orlando tangelo	*C. reticulata* Blanco	43	Washington navel orange		*C. sinensis* (L.) Osbeck
21	Sunburst tangerine	Robinson mandarin × Osceola mandarin		44	Jaffa		*C. sinensis* (L.) Osbeck
22	Okitsu*		*C. unshiu* Marcovitch	45	Pineapple		*C. sinensis* (L.) Osbeck
23	Ovari		*C. unshiu* Marcovitch	46	Trovita		*C. sinensis* (L.) Osbeck
47	Shamouti		*C. sinensis* (L.) Osbeck	74	Ray Ruby		*C. paradisi* Macfadyen
48	Kodur Sathgudi		*C. sinensis* (L.) Osbeck	75	Red Blush		*C. paradisi* Macfadyen
49	Valencia*		*C. sinensis* (L.) Osbeck	76	Rio Red*		*C. paradisi* Macfadyen
50	Campbell Valencia		*C. sinensis* (L.) Osbeck	77	Star Ruby		*C. paradisi* Macfadyen
51	Mid Knight Valencia		*C. sinensis* (L.) Osbeck	78	Foster (Foster pink)		*C. paradisi* Macfadyen
52	Olinda old		*C. sinensis* (L.) Osbeck	79	Marsh	Chance seedling	*C. paradisi* Macfadyen
53	Cutter Valencia		*C. sinensis* (L.) Osbeck	80	Duncan		*C. paradisi* Macfadyen
54	Delta Valencia		*C. sinensis* (L.) Osbeck	81	RGC-9		
55	Lane late		*C. sinensis* (L.) Osbeck	82	RGC-7		
56	Rodhe Red		*C. sinensis* (L.) Osbeck	83	RGC-1		
57	Early gold	Citrus × aurantium L	*C. sinensis* (L.) Osbeck	84	Ches White Pummelo		*C. maxima* (Burm.) Merr
58	Itaboria		*C. sinensis* (L.) Osbeck	85	Devanahalli		*C. maxima* (Burm.) Merr
59	Westin		*C. sinensis* (L.) Osbeck	86	Ches Pink Pummelo		*C. maxima* (Burm.) Merr
60	Mosambi		*C. sinensis* (L.) Osbeck	87	Pink Pummelo*		*C. maxima* (Burm.) Merr
61	Phule Mousambi		*C. sinensis* (L.) Osbeck	88	White Pummelo		*C. maxima* (Burm.) Merr
62	M-3		*C. sinensis* (L.) Osbeck	89	Seed less white (Pummelo)		*C. maxima* (Burm.) Merr
63	M-4		*C. sinensis* (L.) Osbeck	90	Seed star Pummelo		*C. maxima* (Burm.) Merr
64	M-8		*C. sinensis* (L.) Osbeck	91	NRCC P-1	Clone of pummelo	*C. maxima* (Burm.) Merr
65	Crescent orange		*C. sinensis* (L.) Osbeck	92	NRCC P-2	Clone of pummelo	*C. maxima* (Burm.) Merr
66	Fucumoto Marvel		*C. sinensis* (L.) Osbeck	93	NRCC P-3	Clone of pummelo	*C. maxima* (Burm.) Merr
67	Vernia		*C. sinensis* (L.) Osbeck	94	NRCC P-4	Clone of pummelo	*C. maxima* (Burm.) Merr
68	Blood red		*C. sinensis* (L.) Osbeck	95	NRCC P-5	Clone of pummelo	*C. maxima* (Burm.) Merr
69	Fischier			96	PTF-1	Clone of pummelo	*C. maxima* (Burm.) Merr
70	Tinsula sweet orange			97	PTF-2	Clone of pummelo	*C. maxima* (Burm.) Merr
71	RGC-4			98	PTF-3	Clone of pummelo	*C. maxima* (Burm.) Merr
72	Ruby Red			99	PTF-4	Clone of pummelo	*C. maxima* (Burm.) Merr
73	Flame grapefruit		*C. paradisi* Macfadyen	100	Chakotra Local		*C. maxima* (Burm.) Merr
101	Muscat Pummelo		*C. maxima* (Burm.) Merr	126	Katazamir		*C. jambhiri* Lush
102	Tinshukhia	Pummelo × Sweet orange hybrid		127	Chetali Rough lemon		*C. jambhiri* Lush
103	Gou Tou Cheng		Citrus × aurantium	128	Thailand Rough lemon		*C. jambhiri* Lush
104	Marmalade	*Citrus maxima* and *Citrus reticulata*	*C. aurantium L*	129	Florida Rough Lemon		*C. jambhiri* Lush
105	South Africa Sour Orange		*C. aurantium L*	130	Brazil Rough lemon		
106	Smooth Flat Seville	*C. maxima* × *C. reticulata*	*C. aurantium L*	131	Abohar Rough lemon		
107	Sour orange*		*C. aurantium*	132	Baduvapuli lemon	NBPGR, New Delhi, India	
108	Mexican Lime	micrantha × citron	*C. aurantifolia* (Christm.) Swingle	133	South African Rough lemon		
109	Persian Lime		*C. latifolia* (Yu. Tanaka) Tanaka	134	Rubidoux*		*P. trifoliata* (L.) Raf
110	Bears Lime		*C. latifolia* (Yu. Tanaka) Tanaka	135	Chetali trifoliate orange		*P. trifoliata* (L.) Raf
111	Lisbon lemon			136	Gonicoppal trifoliate		*P. trifoliata* (L.)Raf
112	Kagzi Lime*		*C. aurantifolia* Balanco	137	Swingle citrumelo	Duncan grapefruit × *P. trifoliata*	X Citroncirus spp. RUTACEAE
113	Sweet Lime*		*C. limettioides*	138	Sacaton citrumelo	*P. trifoliata* × *C. paradisi*	X Citroncirus spp.
114	Baramasi Lemon*		*C. limon* (L.) Burm	139	Carrizo	'Washington' sweet orange × *P. trifoliata*	X Citroncirus sp. RUTACEAE
115	Eureka lemon	sour orange × citron	*C. limon* (L.) Burm	140	Kurshashke citrange		
116	Indian meyer lemon	Citrus × meyeri	*C. limon* (L.) Burm	141	X-639	Cleopatra mandarin × *P. trifoliata*	X Citroncirus spp. RUTACEAE
117	Limoneriaassam			142	C-32	Ruby' orange x 'Webber-Fawcett' trifoliate	X Citroncirus spp.
118	Baramasi lemon		*C. limon* (L.) Burm	143	Benton	Ruby Blood sweet orange × *P. trifoliata*	X Citroncirus spp. RUTACEAE
119	Bhardi lemon			144	C-35 J	Ruby' orange x 'Webber-Fawcett' trifoliata	X Citroncirus spp.
120	Rough lemon local*		*C. jambhiri* Lush	145	U- 852	*C. reticulate* ‘Changsha’ × *P. trifoliata*	
121	Schaub Rough lemon		*C. jambhiri* Lush	146	Rich 16–6		*P. trifoliata* (L.) Raf
122	Hayer Rough Lemon		*C. jambhiri* Lush	147	NRCC 1	Rough lemon × Troyer citrange	
123	14–9-13 Rough lemon		*C. jambhiri* Lush	148	NRCC 3	Rough lemon × Troyer citrange	
124	Florida Rough lemon		*C. jambhiri* Lush	149	NRCC 4	Rough lemon × trifoliate orange	
125	Karna khatta Rough lemon		*C. kharna*	150	NRCC 5	Rough lemon × trifoliate orange	
151	Norneo Rangpur lime		*C. limonia* osbeck	167	Narangi (Garden)		
152	Nornia Rangpur lime		*C. limonia* osbeck	168	RGC-2		
153	Texas Rangpur Lime		*C. limonia* osbeck	169	Miami Kumquat		*Fortunella margarita* (Lour.)
154	Rangpur Lime J*			170	Alemow*	*C. macrophylla*	*C. macrophylla* Wester
155	Chetalli Rangpur Lime		*C. limonia* osbeck	171	Indian wild orange*	*C. indica*	*C. indica*
156	Brazalian Rangpur Lime		*C. limonia* osbeck	172	Citron*		*C. macroptera*
157	South African Rangpur lime		*C. limonia* osbeck	173	Etrog*		*C. medica* L
158	Gonicoppal Rangpur Lime		*C. limonia* osbeck	174	Cleminula species (clementina)		
159	Australia Sour trifoliate			175	Sadaphal species		
160	Volkameriana	Sour mandarin × citron	*C. volkameriana*	176	Gajanima species		
161	Cleopatra*	Open pollinated seedling	*C. reshni* Hort.ex Tanaka	177	Calamondin*	(Fortunella × *C. reticulata*	*C. madurensis* Lour
162	Pectinifera		*C. depressa*	178	RGC-3		
163	San Chu Sha*		*C. reticulata* Blanco	179	RGC-5		
164	Kinkoji	Graft hybrid of *C. obovoidea* + Satsuma	*C. obovoidea* Takahashi	180	RGC-6		
165	Chinotto*		*C. myrtifolia* Rafinesque	181	RGC-8		
166	Fortunella*		*Fortunella hindsii* Champ. Ex Benth				

PCR amplification was done for wet-lab using final volume 10 µL reaction mixture (2.5 mM Taq buffer, 1.5 mM MgCl_2_, 0.2 mM deoxynucleotide triphosphate (dNTPs), 0.4 µM primer, and 1.0 U of Taq DNA polymerase) using Thermo scientific ABI thermocycler. The amplification was achieved using a thermal PCR profile of initial denaturation at 94 ˚C for 5 min, followed by 35 cycles of denaturation at 94 ˚C for 1 min, annealing at varied from primer to primer for 1.30 min and extension at 72 ˚C for 1.30 min, and a final extension at 72 ˚C for 7 min. PCR products were separated on 3.5% molecular grade agarose gel (VWR, Life Science, India), and visualized under UV light in gel documentation and the amplicons were scored on Alpha Innotech Alpha Imager Hp System(SYNGENE, G: Box, USA). The amplified DNA fragments for all primers were scored as ‘1’ for presence or ‘0’ for the absence and base pairs size of each fragment in all studied genotypes.

### Population structure and phylogeny analysis

The genotypic data were generated from 24 initially tested germplasm and finally 181 tested germplasm and generated data were utilized for assessing the genetic variability parameters through GenAlEx v. 6.5 software^89^, the TP, TPP, N, Na, I, MAF, Ne, Ho, He, uHe, and PIC. A dendrogram was generated on the basis of the distance matrix using an unweighted pair group with arithmetic mean (UPGMA) based cluster analysis and principal coordinate analysis (PCoA) through DARwin v. 6.0.021 software for studying the genetic relatedness among genotypes^93^.

STRUCTURE v.2.3.441 was used to estimate population structure using Bayesian clustering. The admixture ancestry and correlated allele frequency model were used to perform structure analysis for K (number of subpopulations, five separate runs with a burn-in length of 100,000 and MCMC repetitions of 100,000 were done for each K) values ranging from 1 to 10. The optimal K was calculated through delta K estimation method 42 by using STRUCTURE Harvester43. Citrus germplasm was divided into sub-populations depending on the probability of cluster assignment (Q). To allocate citrus accessions to each group, the cluster assignment probability (Q) value of 0.50 was employed.

### Functional gene annotation

BLASTX was used to examine the flanking regions against the GenBank non-redundant protein database to assign probable functions of the discovered SSR marker. To assign putative functions to each locus, the best matched sequences with P < 0.001 were utilised, and the putative functions were saved in a text file. A Blast2Go analysis was used to functionally annotate SSR loci.

## Supplementary Information

Supplementary Information 1.

Supplementary Information 2.

Supplementary Information 3.

Supplementary Information 4.

Supplementary Information 5.

Supplementary Information 6.

Supplementary Information 7.

## Data Availability

SSR markers were designed from citrus genome sequences which were retrieved from Data Download|Citrus Genome Database (citrusgenomedb.org). Accession number of all species are available in NCBI viz., *C. sinensis* genome v2.0, HZAU ( PRJNA86123), *C. clementina* genome v1.0, JGI (PRJNA232045), *C. maxima* genome v1.0, HZAU (PRJNA318855), *C. medica* genome v1.0, HZAU (PRJNA320023), *C. ichangensis* genome v1.0, HZAU (PRJNA321657), *A. buxifolia* genome v1.0, HZAU (PRJNA327148), *Fortunella hindsii* acc. S3y‐45 (PRJNA487160). The data analysed during the current study are available in the supplementary Table S2.
