# Supplementary material for: Comprehensive genome-wide identification and transferability of chromosome-specific highly variable microsatellite markers from citrus species
Source: Sci Rep. 2023 Jul 5;13:10919. doi: 10.1038/s41598-023-37024-0 (PMC10322976; doi:10.1038/s41598-023-37024-0)
Supplement: Supplementary file 7 — Supplementary Information 7. [file 41598_2023_37024_MOESM7_ESM.docx]

**Comprehensive genome-wide identification and transferability of chromosome-specific highly variable microsatellite markers from citrus species**

Jagveer Singh^1,2^, Ankush Sharma^3^, Vishal Sharma^4^, Popat Nanaso Gaikwad^1^, Gurupkar Singh Sidhu^1^, Gurwinder Kaur^1^, Nimarpreet Kaur^1^, Taveena Jindal^1^, Parveen Chhuneja^1^ and HS Rattanpal^5^

^1^School of Agricultural Biotechnology, Punjab Agricultural University, Ludhiana 141004, India

**^2^Department of Fruit Science, College of Horticulture & Forestry, Acharya Narendra Deva University of Agricultural & Technology, Kumarganj 224229, India**

^3^Plant Genome Mapping Laboratory, University of Georgia, Athens, Georgia, 30602, USA

^4^National Agri-Food Biotechnology Institute, Sector-81, SAS Nagar, Mohali 140308, Punjab, India

^5^Department of Fruit Science, Punjab Agricultural University, Ludhiana 141004, India


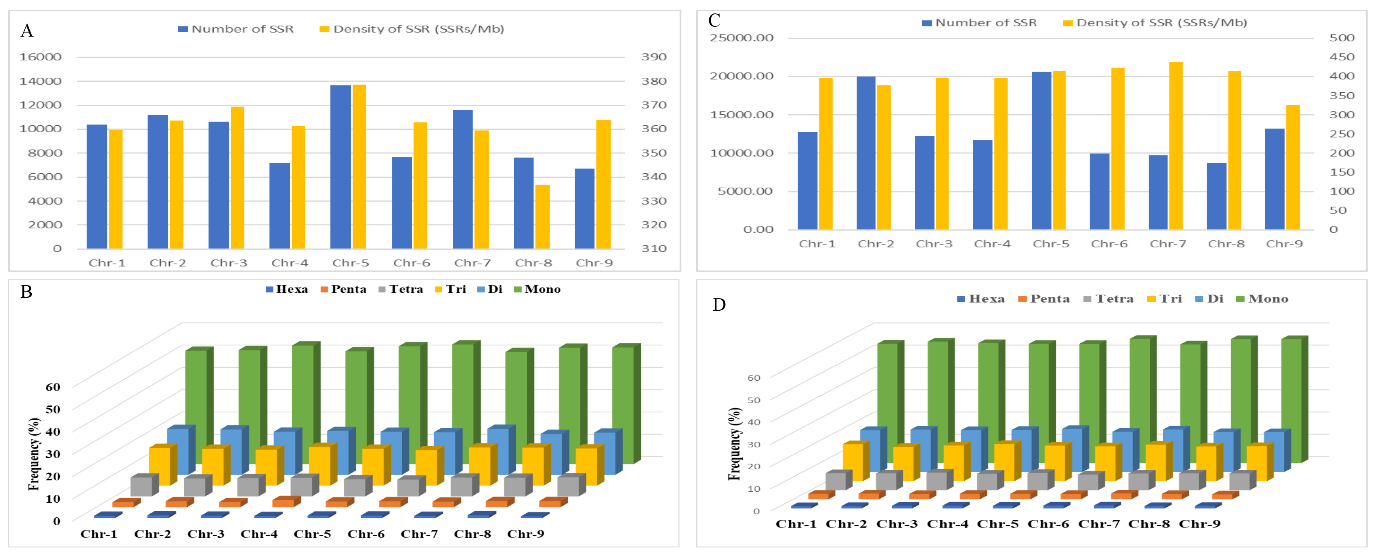


**Supplementary Figure 1.** Comprehensive Characterization and Validation of Chromosome-Specific Highly Polymorphic SSR Markers from *C. sinensis* (A, B) and *C. maxima* (C, D).


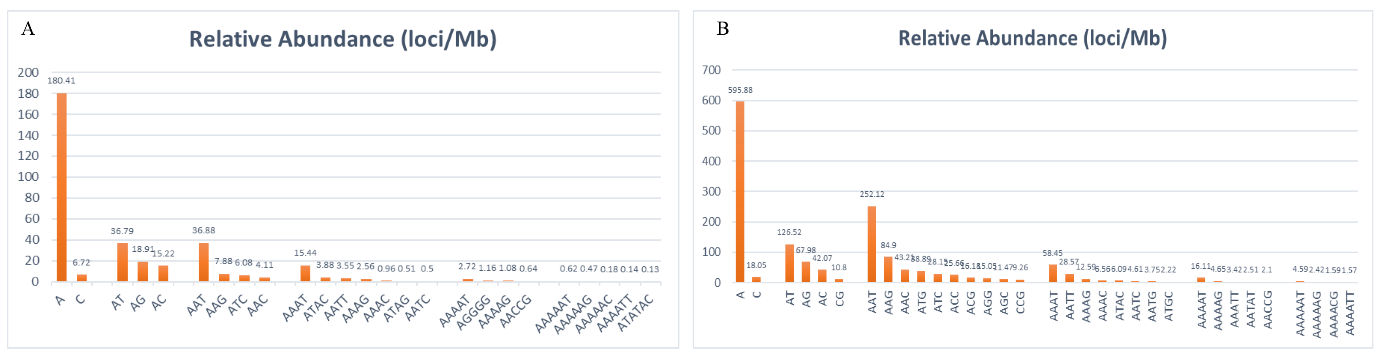


**Supplementary Figure 2.** Comprehensive Characterization and Validation of Chromosome-Specific Highly Polymorphic SSR Markers from *C. sinensis* (A) and *C. maxima* (B).


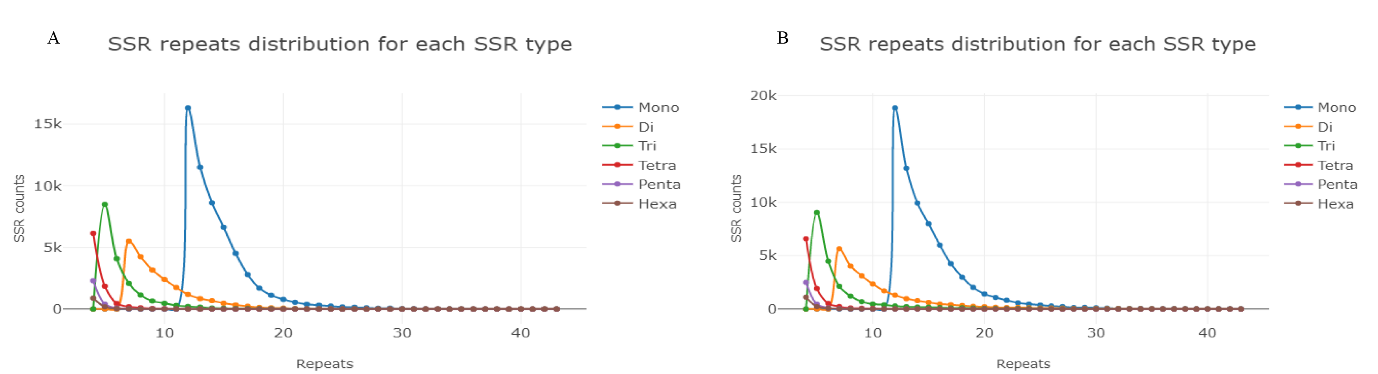


**Supplementary Figure 3.** Comprehensive Characterization and Validation of Chromosome-Specific Highly Polymorphic SSR Markers from *C. sinensis* (A) and *C. maxima* (B).


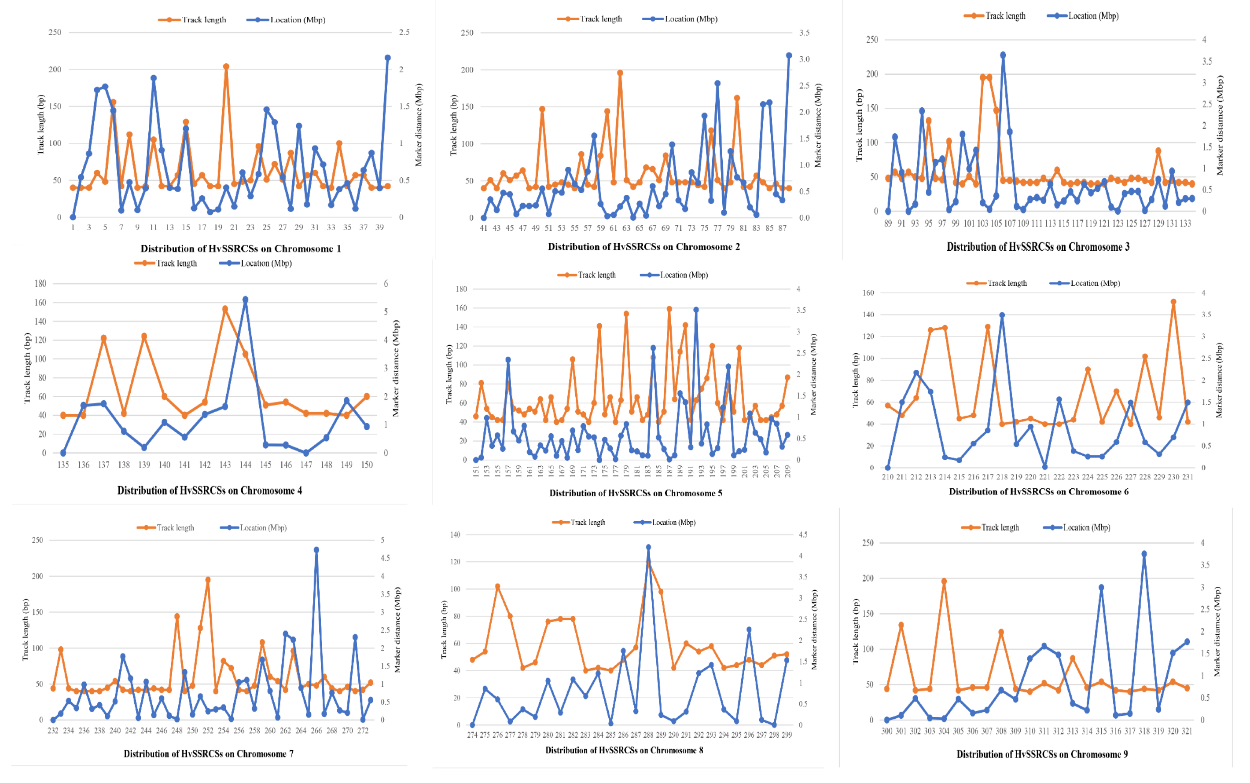


**Supplementary Figure 4A.** Comprehensive Characterization and Validation of Chromosome-Specific Highly Polymorphic SSR Markers from *C. sinensis.*


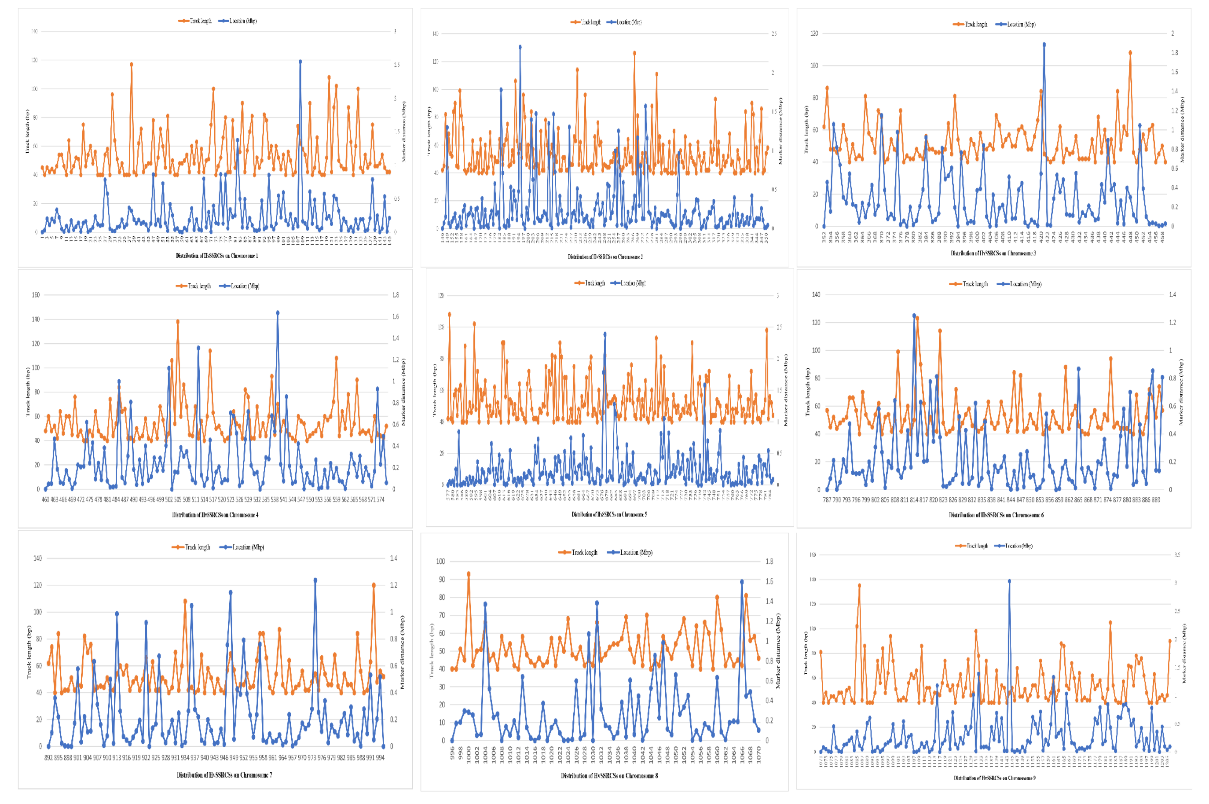


**Supplementary Figure 4B.** Comprehensive Characterization and Validation of Chromosome-Specific Highly Polymorphic SSR Markers from *C. maxima.*


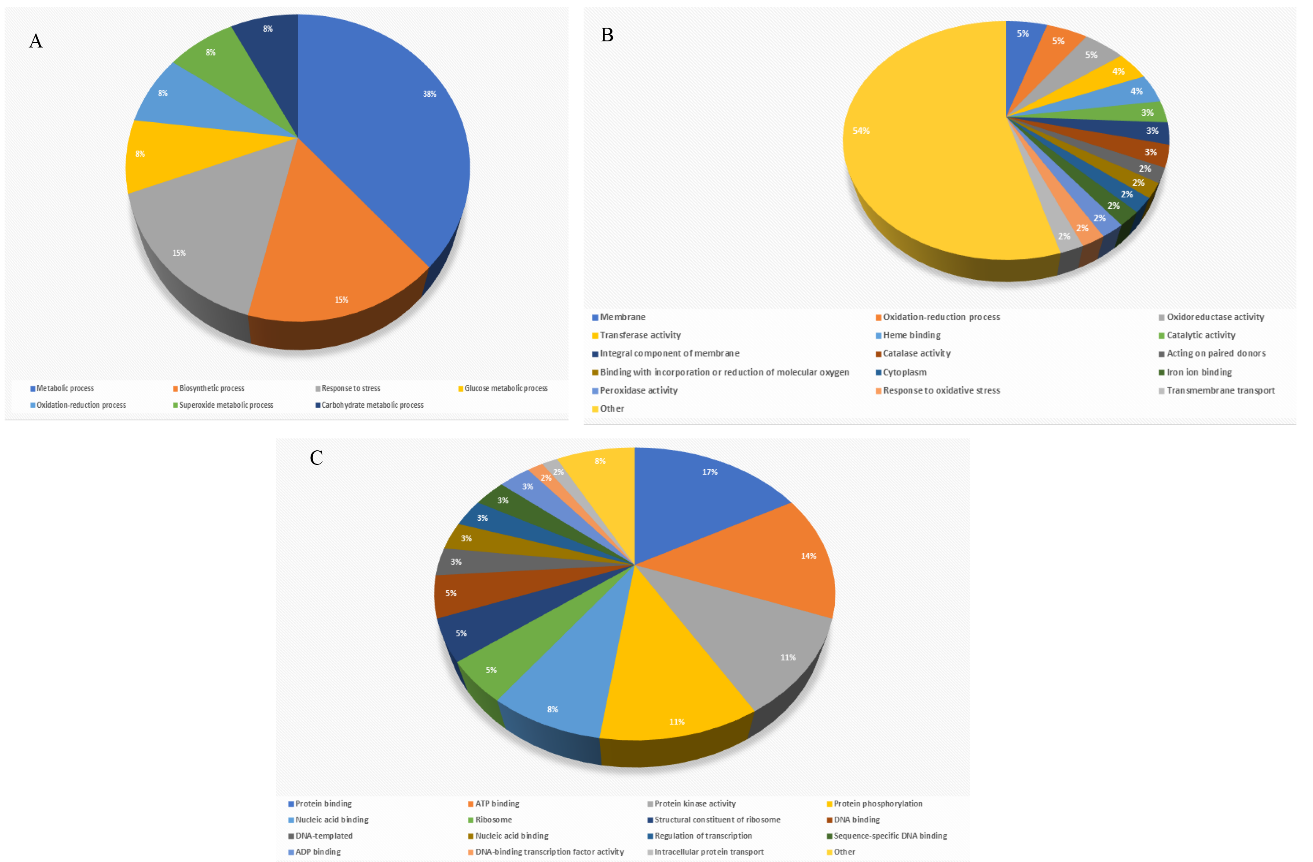


**Supplementary Figure 5A.** Gene Ontology of genome wide developed *C. sinensis* SSR marker and their flanking regions, GO biological process (A), molecular function (B) and cellular component (C).


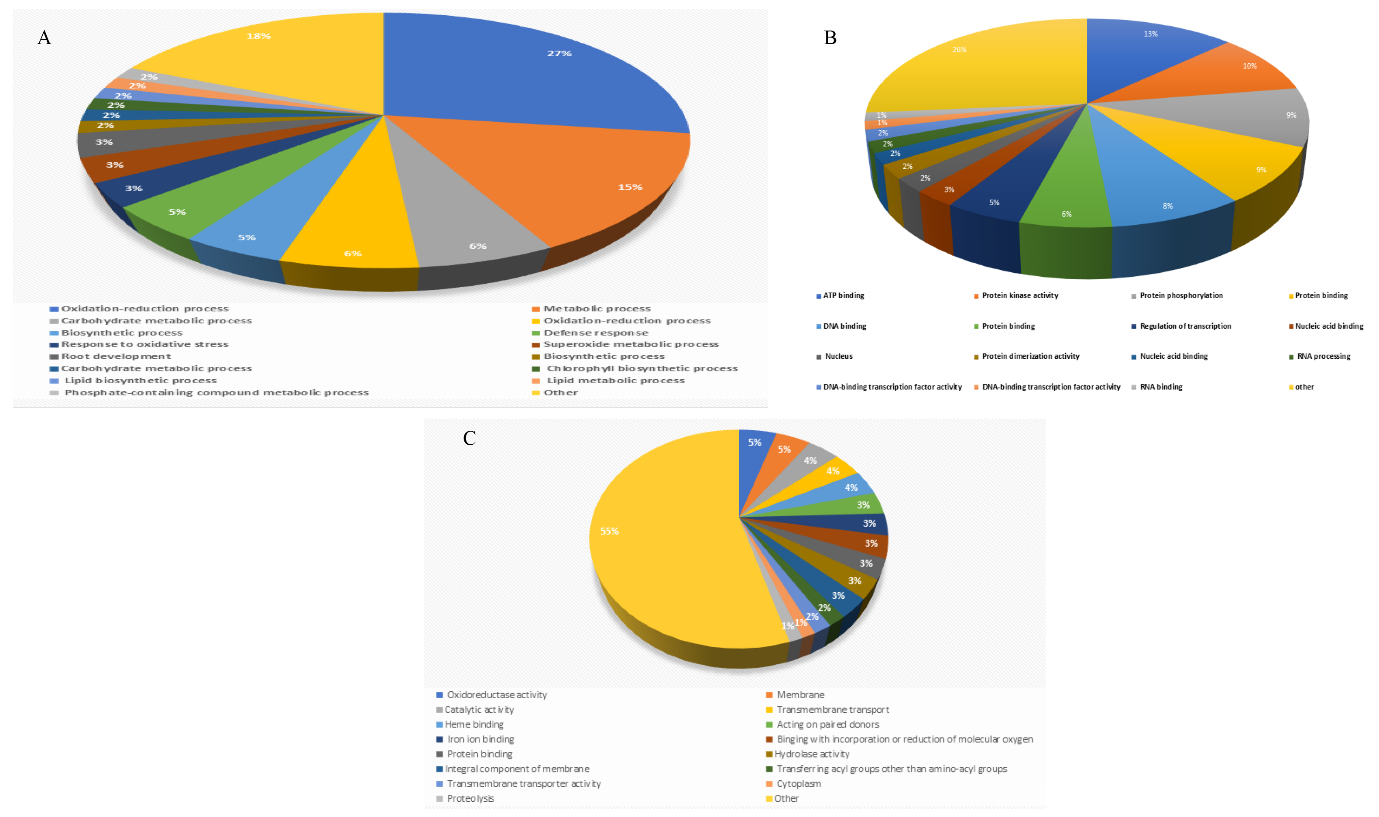


**Supplementary Figure 5B.** Gene Ontology of genome wide developed *C. maxima* SSR marker and their flanking regions, GO biological process (A), molecular function (B) and cellular component (C).
